# Crucial Role of Juvenile Hormone Receptor Components Methoprene-Tolerant and Taiman in Sexual Maturation of Adult Male Desert Locusts

**DOI:** 10.3390/biom11020244

**Published:** 2021-02-09

**Authors:** Michiel Holtof, Joachim Van Lommel, Marijke Gijbels, Elfie Dekempeneer, Bart Nicolai, Jozef Vanden Broeck, Elisabeth Marchal

**Affiliations:** 1 Department of Biology, Molecular Developmental Physiology and Signal Transduction Lab., Division of Animal Physiology and Neurobiology, Naamsestraat 59-Box 2465, B-3000 Leuven, Belgium; michiel.holtof@kuleuven.be (M.H.); joachim.vanlommel@kuleuven.be (J.V.L.); marijke.gijbels@kuleuven.be (M.G.); Elisabeth.Marchal@imec.be (E.M.); 2 Division of Mechatronics, Biostatistics and Sensors (MeBioS), KU Leuven, Willem de Croylaan 42-Box 2428, B-3001 Leuven, Belgium; elfie.dekempeneer@kuleuven.be (E.D.); bart.nicolai@kuleuven.be (B.N.)

**Keywords:** accessory gland, *corpora allata*, courtship, insect, Krüppel-homolog 1, pheromone, testis

## Abstract

Currently (2020), Africa and Asia are experiencing the worst desert locust (*Schistocerca gregaria*) plague in decades. Exceptionally high rainfall in different regions caused favorable environmental conditions for very successful reproduction and population growth. To better understand the molecular mechanisms responsible for this remarkable reproductive capacity, as well as to fill existing knowledge gaps regarding the regulation of male reproductive physiology, we investigated the role of methoprene-tolerant (*Scg*-Met) and Taiman (*Scg*-Tai), responsible for transducing the juvenile hormone (JH) signal, in adult male locusts. We demonstrated that knockdown of these components by RNA interference strongly inhibits male sexual maturation, severely disrupting reproduction. This was evidenced by the inability to show mating behavior, the absence of a yellow-colored cuticle, the reduction of relative testes weight, and the drastically reduced phenylacetonitrile (PAN) pheromone levels of the treated males. We also observed a reduced relative weight, as well as relative protein content, of the male accessory glands in *Scg*-Met knockdown locusts. Interestingly, in these animals the size of the *corpora allata* (CA), the endocrine glands where JH is synthesized, was significantly increased, as well as the transcript level of JH acid methyltransferase (JHAMT), a rate-limiting enzyme in the JH biosynthesis pathway. Moreover, other endocrine pathways appeared to be affected by the knockdown, as evidenced by changes in the expression levels of the insulin-related peptide and two neuroparsins in the fat body. Our results demonstrate that JH signaling pathway components play a crucial role in male reproductive physiology, illustrating their potential as molecular targets for pest control.

## 1. Introduction

Reproductive physiology in animals is strictly regulated by hormones. However, depending on the species, these hormones and their specific contributions may differ. This is illustrated nicely by reports on the endocrine regulation of the female reproductive physiology in a wide variety of insect species (as reviewed by [1]). Two important players in this process are the sesquiterpenoid, juvenile hormone (JH), and the ecdysteroid, 20-hydroxyecdysone (20E). Both lipophilic hormones are also key in the control of larval development and metamorphosis of insects. Whereas 20E triggers successive molts throughout the insect’s life cycle, JH, as its name indicates, represses metamorphosis and thus keeps the insect in a juvenile stage. Ecdysone is synthesized in the prothoracic glands of juvenile insects. These glands degenerate in adult insects and ecdysteroid synthesis takes place in the reproductive system [2,3,4]. JH on the other hand is produced in the *corpora allata* (CA), small paired endocrine glands situated in the head [3,4,5]. Important enzymes catalyzing the final steps of JH biosynthesis in the desert locust’s CA are juvenile hormone acid O-methyltransferase (JHAMT) and methyl farnesoate epoxidase (CYP15A1) [6].

The 20E signaling pathway with its heterodimeric receptor complex, consisting of ecdysone receptor (EcR) and retinoid X receptor/ultraspiracle (RXR/USP), and downstream transcription factors, many belonging to the nuclear receptor superfamily, has been widely researched [7]. However, for a long time the JH signaling pathway remained elusive. Methoprene-tolerant (Met), a member of the basic-helix-loop-helix (bHLH)/Per-Arnt-Sim (PAS) family of transcription factors, was first described in 1986 as a crucial factor in the resistance to the commercially available JH analog, Methoprene [8]. The possibility that Met might act as a JH receptor was later confirmed by Miura *et al*., who showed that the recombinant *Drosophila melanogaster* Met protein can bind JH with high affinity [9]. The study by Charles et al. later showed that a *Tribolium castaneum* Met contains a conserved hydrophobic binding pocket within the PAS-B domain, which binds JH and its analogs with high affinity, and that this capacity is necessary for interaction of Met with its partner, another member of the bHLH-PAS family named SRC (homologous to the mammalian steroid receptor coactivator 1) or Taiman (Tai), following FlyBase nomenclature [10]. Tai had previously been shown in yeast two-hybrid studies to interact with Met [11,12]. Further downstream of the Met-Tai complex, the JH signal was later found to be transduced by Krüppel-homolog 1 (Kr-h1), a C2H2 zinc-finger containing transcription factor [13].

In various insect species, RNA interference (RNAi) based reverse genetics studies showed that Met, its binding partner Tai, and their downstream transcription factor Kr-h1 are indeed transducing the anti-metamorphic JH signal [14,15,16,17,18,19,20]. Key regulators of metamorphosis are often referred to as the MEKRE93 pathway, composed of the JH receptor Met and the transcription factors Kr-h1 and E93. Expression of E93, a key determinant promoting adult morphogenesis, is repressed by Kr-h1 during larval-to-larval transitions but will rise once JH titers drop and Kr-h1 expression is lowered before the onset of metamorphosis [21,22,23]. Studies on Met, Tai and Kr-h1 were further elaborated towards other processes in which JH was reported to play a role, such as the developmental maturation of the female reproductive system and the reproductive diapause that is observed in a variety of insect species [24,25,26,27,28,29,30,31]. A recent study in the migratory locust, *Locusta migratoria*, has shown that one isoform of Tai contains a PRD-repeat motif that is essential for the induction of vitellogenesis by JH [32]. Until now, most research on the reproductive physiology of insects has focused on the role of these JH pathway components in females, while their exact role in males is much less understood.

Therefore, in the current study we decided to focus on the role of the JH receptor components Met and Tai in the reproductive maturation of males of the desert locust, *Schistocerca gregaria*. Maturation in crowd-reared adult male *S. gregaria* is associated with the display of sexual behavior and the bright yellow coloration of the cuticle due to the accumulation of carotenoids [33,34]. The role of JH in male locusts has previously been studied by allatectomy (physical removal of the CA or chemical apoptosis of the CA cells using precocene) and JH (analog) treatments [35,36]. In this regard, Amerasinghe published an interesting study showing that JH can rescue the yellow coloration of the cuticle, as well as the production of maturation pheromone emissions, in allatotectomized male *S. gregaria* [37]. Phenylacetonitrile (PAN) later turned out to be a critical volatile in the pheromone bouquet of sexually mature gregarious male desert locusts [38,39,40]. PAN acts as a courtship inhibiting pheromone with which sexually mature males can ensure postcopulatory mate guarding [41]. Although JH levels correlate well with PAN production in adult males, JH is suggested to regulate PAN biosynthesis only indirectly by stimulating the development of pheromone producing tissues [42,43]. Furthermore, a study by Braun and Wyatt has shown that JH is necessary for the growth of the male accessory glands (AG) and for the associated protein synthesis in the fat body [44]. The insect fat body plays a pivotal role in many metabolic processes, including nutrient and energy homeostasis, as well as reproduction [45]. A clear correlation between the JH biosynthetic activity of the CA and the dry weight of the AG was also found in a study by Avruch and Tobe, who additionally showed a direct relationship between rates of JH release from the CA and the age of the animals [46]. In the migratory grasshopper, *Melanoplus sanguinipes*, the biosynthesis of specific proteins in the AG is similarly highly dependent on JH [47].

Adequate nutrient uptake and allocation are pivotal for successful reproductive development in insects [48]. In general, the insulin/insulin-like growth factor (IGF) signaling pathway (ISP), sensing the organism’s nutrient status and positively controlling vitellogenesis and oocyte growth in female insects, plays a crucial role in the trade-off between reproduction and longevity. Different studies have highlighted the complex interplay between JH and ISP in female reproductive physiology [49,50]. Our previous studies in adult female desert locusts also illustrated this interplay. RNAi-mediated knockdown of *Scg-Met* resulted in a significant increase in relative transcript levels of two neuroparsins (*Scg-NP3* and *Scg-NP4*) in the fat body, while *Scg-IRP* (insulin-related peptide) transcript levels decreased, suggesting a consistent cross-talk between JH signaling via *Scg*-Met and *Scg*-NP and *Scg*-IRP expression [14]. In line with this, knockdown of *Scg*-IRP in female *S. gregaria* was shown to reduce vitellogenin (*Vg*) transcript levels and oocyte growth [51]. This study also showed that knocking down the *S. gregaria* NPs, in contrast to *Scg*-IRP, increased *Scg-Vg* transcript levels and resulted in larger oocytes. NPs were initially characterized as antigonadotropic factors from the *pars intercerebralis*-*corpora cardiaca* (CC) neurohemal complex of locusts [52,53,54]. They belong to a large family of cysteine-rich secreted proteins, which also includes the ovary ecdysteroidogenic hormone (OEH), a gonadotropic factor in blood-fed mosquitoes, and shows sequence similarities with the most conserved region of vertebrate insulin-like growth factor binding proteins (IGFBPs) [55,56,57]. While in mosquitoes the gonadotropic OEH has been shown to act in parallel with the ISP via a Venus flytrap containing receptor tyrosine kinase, the exact mechanism of in vivo action of the antigonadotropic *Scg*-NPs remains unknown [58,59]. Additionally, in male insects ISP has been reported as a nutrient-dependent regulator of reproductive physiology, acting at the level of spermatogenesis, spermatocyte growth and reproductive dimorphism, but this has received much less attention so far (as reviewed by [48]).

In this paper, we report on the systemic RNAi knockdown of the JH receptor components in adult *S. gregaria* males. This experimental treatment significantly inhibited sexual maturation of male locusts, indicating that the molecular components of the JH receptor complex are crucial for this process. Our study shines new light on the molecular patterns of male desert locust maturation, a promising, but as yet largely neglected field of study in the search towards novel strategies to control devastating locust plagues.

## 2. Materials and Methods

### 2.1. Animal Rearing

Desert locusts were reared under crowded conditions in large cages, in which temperature (32 °C), relative humidity (40–60%) and light exposure (13 h photophase) were controlled. The animals were fed daily with dry oat flakes and fresh cabbage. Following mating, mature females were allowed to deposit their eggs in pots filled with damp sand. Each week, these pots were collected and transferred to empty cages, where eggs were allowed to hatch. In the described experiments, fifth nymphal and adult locusts were collected at the time of ecdysis and transferred to separate cages to obtain cohorts of temporally synchronized animals.

### 2.2. In Silico Analysis of JH Signaling Components

#### 2.2.1. Amino Acid Sequence Predictions of *Scg*-Met and *Scg*-Tai

Partial cDNA sequences for *Scg-Met* and *Scg-Tai* were initially found in an in-house whole-body transcriptome database of *S. gregaria.* Both partial cDNA sequences were uploaded to NCBI Genbank, with accession numbers MK855050 (*Scg-Met*) [14], and MK442071 (*Scg-Tai*). To further identify putative full-length isoforms of *S. gregaria* Met and Tai, several BLAST searches were performed in the recently published *S. gregaria* genome database [60] using publicly available (NCBI) sequences of Methoprene-tolerant and Taiman, as well as the above-mentioned partial desert locust cDNAs. The *S. gregaria* genome data are available on ORCAE (https://bioinformatics.psb.ugent.be/orcae/overview/Schgr (accessed on 8 January 2021)). Candidate nucleotide sequences were translated to the best candidate coding sequences (CDS) by Transdecoder. Coding regions were (1) scanned (HMMER) in the Pfam database, (2) scanned for potential signal peptide sequences with SignalP, (3) scanned for potential transmembrane domain sequences with TMHMM. This enabled us to extend the initial *Scg*-Met and *Scg*-Tai amino acid sequence predictions (MK855050 and MK442071) with the genome sequence derived predictions that are publicly available on ORCAE (SCHGR_00003583 and SCHGR_00005654, respectively).

#### 2.2.2. Multiple Sequence Alignments

Protein sequences of Met and Tai orthologs from several other insect species were obtained from NCBI (Table S3). Selected protein sequences were aligned using the Clustal Omega algorithm and visualized using Mview [61]. Conserved protein domains were identified using NCBI conserved domain search [62]. A maximum likelihood phylogenetic analysis was performed on this multiple sequence alignment using IQTREE (version 1.6.12, VT+F+I+G4, 1000 SH-aLRT replicates and 1000 ultrafast bootstrap replicates [63]. Phylogenetic trees were visualized using iTOL [64].

### 2.3. RNA Interference

#### 2.3.1. Production of dsRNA

dsRNA constructs for *Scg-Met* (*dsScg-Met*) and *Scg-Tai* (*dsScg-Tai*) were prepared using the MEGAscript^®^ RNAi Kit (ThermoFisher Scientific, Waltham, MA, USA), designed for the production of dsRNAs longer than 200 bp, according to the manufacturer’s instructions. The procedure is based on the high-yield in vitro transcription reaction catalyzed by T7 RNA Polymerase starting from a user-provided linear template DNA flanked by T7 promoter sites. The dsRNA is obtained by annealing the resulting sense and antisense RNA strands. Primer sequences used for obtaining the linear templates are given in Table S1. A PCR with REDTaq^®^ DNA polymerase (Sigma-Aldrich, Saint Louis, MO, USA) was performed and the length of the amplified products was checked by 1% agarose gel electrophoresis. These bands were further cloned and sequenced (TOPO^®^ TA cloning kit for sequencing, Invitrogen) to confirm the amplicon sequence (Sanger sequencing, LGC Genomics, Berlin, Germany). For the production of GFP dsRNA (*dsGFP*), a PCR fragment flanked by a T7 promoter site was cloned in both sense and antisense direction in a TOPO 4.1 sequencing vector (Life Technologies, Carlsbad, CA, USA) and subsequently used as template for in vitro transcription. The final concentration of the produced dsRNA was estimated using a NanoDrop ND-1000 UV-VIS Spectrophotometer, and 1% agarose gel electrophoresis was performed to assess the integrity of the dsRNA.

#### 2.3.2. RNAi Experiment

Adult male locusts were injected with 1 µg dsRNA (in 6 µL *S. gregaria* Ringer solution) targeting *Scg-Met* (*n* = 25) or *Scg-Tai* (*n* = 20) (1 L Ringer solution: 8.766 g NaCl; 0.188 g CaCl_2_; 0.746 g KCl; 0.407 g MgCl_2_; 0.336 g NaHCO_3_; 30.807 g sucrose; 1.892 g trehalose; pH 7.2). For all injections, the needle of the micro-syringe (Hamilton Company, Reno, NV, USA) was inserted laterally between the second and third abdominal segments and oriented towards the animal’s anterior side. The corresponding control groups were injected with 1 µg *dsGFP* (*n* = 25 in *dsScg-Met* experiment and *n* = 20 in *dsScg-Tai* experiment). Injections were initiated at the first day after the adult molt (day 0) and were repeated every 3 days to ensure a potent and persistent knockdown of the targets. The injections were pursued until day 34 after the adult molt. Then animals were sacrificed to collect their testes, AG, CA, fat body, and epidermis (originating from the ventral side of the third abdominal segment).

### 2.4. Observation of Mating Behavior

During the RNAi experiments, males from the same treatment condition [*dsScg-Met* (*n* = 25), *dsGFP* (*n* = 25 in *dsScg-Met* experiment), *dsScg-Tai* (*n* = 20) and *dsGFP* (*n* = 20 in *dsScg-Tai* experiment) treatments] were housed together in treatment-specific cages. From the day of the first injection, the first day after the adult molt (day 0), equal numbers of untreated female desert locusts of the same age were added to these cages to monitor occurrence of mating behavior. Mating behavior was observed during the following 34 days. Then treated males were sacrificed as described in Section 2.3.

### 2.5. Calculation of the Accessory Gland Somatic and Gonadosomatic Indices

On day 34 of the adult stage, the live animals, as well as their dissected accessory glands (AG) and testes, were weighed using a Sartorius WME5004-e04112 balance. The accessory gland somatic (AGSI) and gonadosomatic (GSI) indices were calculated as the ratio of the AG and testes weight to the whole-body weight, respectively. The protein content of the AG was determined using the bicinchoninic acid assay (BCA) method and normalized to the respective AG weight.

### 2.6. Corpora Allata Surface Area Measurement

The CA were carefully dissected and cleaned in *S. gregaria* Ringer solution and transferred to a small petri dish. Images of the CA were obtained using a light microscope (Zeiss SteREO Discovery.V8) (Carl Zeiss, Oberkochen, Germany) equipped with an AxioCam ICc3 camera using the AxioVision 4.7 (Carl Zeiss, Oberkochen, Germany). These images were further processed in ImageJ [65] allowing the surface area measurement of the CA in the image which was normalized to the locust’s total body weight.

### 2.7. Volatile Determination

On day 26 of the adult stage, live locusts [*dsScg-Met* (*n* = 5) and *dsGFP* control (*n* = 5); *dsScg-Tai* (*n* = 4) and *dsGFP* control (*n* = 4)] were analyzed for aroma. Individual locusts were introduced into 60 mL screw neck headspace vials (Macherey-Nagel, Dueren, Germany). The vial was sealed with TPFE 3.2 mm Beige screw cap (Marcherey-Nagel). The vial was incubated at 40 °C for 60 min. Then, volatiles were extracted with a DVB-CAR-PDMS fiber (Sigma-Aldrich) at 40 °C for 60 min. Determination of volatile compounds was performed on an Agilent 7890 A gas chromatograph (GC) (Agilent Technologies, Santa Clara, CA, USA) coupled to an Agilent 5975C VL MSD Mass Selective Detector (MS) (Agilent Technologies, Santa Clara, CA, USA) and equipped with a Gerstel Multipurpose Sampler 2 (Gerstel GmbH & Co.KG, Mülheim an der Ruhr, Germany). After extraction, aroma compounds were thermally desorbed into the injector heated at 220 °C and equipped with a SPME liner (0.75 i.d., Sigma-Aldrich). Split injection was performed in splitmode of 1:100 and the fiber thermally conditioned for 5 min. Separation was done on an HP-5MS column (30 m × 0.25 mm i.d. × 0.25 μm df) using helium as the carrier gas with a constant flow rate of 1 mL min^−1^. The column oven temperature program was as follows: 40 °C (4 min), 240 °C (10 °C min^−1^) and hold 240 °C (10 min). The total GC run time was 34 min. Mass spectra in the 35 to 350 m/z range were recorded at a scanning speed of 4.17 scan cycles per second. Chromatograms and mass spectra obtained from the GC-MS were deconvoluted and analyzed using MSD Chemstation (Agilent Technologies) and the automated mass spectral deconvolution and identification system (AMDIS) software v.2.1 (National Institute of Standards and Technology (NIST), Gaithersburg, Maryland). Aroma volatile compounds were identified by matching with NIST 11 mass spectral library. Volatile composition was compared by using absolute peak areas.

### 2.8. Transcript Profiling

#### 2.8.1. Sample Collection

Tissues (testes, AG, CA, fat body, and epidermis (originating from the ventral side of the third abdominal segment)) were dissected in *S. gregaria* Ringer solution under a binocular microscope, and immediately transferred to liquid nitrogen to prevent RNA degradation. The dissected tissue samples were stored at −80 °C until further processing prior to RNA extraction. For each tissue and condition, the exact number of biological replicates that were analyzed is indicated in the figure legends.

#### 2.8.2. RNA Preparation

The pooled samples were transferred to MagNA Lyser Green Beads containing tubes and homogenized using a MagNA Lyser instrument (Roche, Basel, Switzerland). Total RNA was subsequently extracted from the tissue homogenate with the RNeasy Lipid Tissue Kit (Qiagen, Hilden, Germany) with additional DNase treatment according to the manufacturer’s instructions. Because of their relatively small size, CA were extracted using the RNAqueous-Micro Kit (Life Technologies, Carlsbad, CA, USA), followed by the recommended DNase step. Quality and concentration of the resulting RNA samples were analyzed using a Nanodrop spectrophotometer (Life Technologies, Carlsbad, CA, USA).

#### 2.8.3. Quantitative Real-Time (q-)RT-PCR

An equal amount of RNA was reverse transcribed into cDNA using the PrimeScript™ RT Reagent Kit, by following the manufacturer’s protocol (Takara, Shiga, Japan). Prior to q-RT-PCR transcript profiling, several previously described housekeeping genes were tested for their stability in the designed experiment [66]. Optimal reference genes were selected using geNorm software [67]. q-RT-PCR primers for reference genes and target genes were designed using Primer Express software (Applied Biosystems, Foster City, CA, USA). For all investigated tissues and conditions, *Scg*-Act and *Scg*-Ef1a were selected by the geNorm software as having the most stable expression and were used as reference genes throughout this study. Primer sets were validated by designing relative standard curves with a serial ten-fold dilution of a calibrator cDNA sample. Efficiency of q-RT-PCR and correlation coefficient (R^2^) were measured for each primer pair. Primers for q-RT-PCR are given in Table S2. All PCR reactions were performed in duplicate in 96-well plates on a StepOne System (ABI Prism, Applied Biosystems, Foster City, CA, USA). Each reaction contained 5 μL Fast Sybr Green, 0.5 μL of Forward and Reverse primers (10 μM), and 4 μL of cDNA. For all q-RT-PCR reactions, the following thermal cycling profile was used: 50 °C for 2 min, 95 °C for 10 min, followed by 40 cycles of 95 °C for 15 s and 60 °C for 60 s. Finally, a melting curve analysis was performed to check for primer dimers. For all transcripts, only a single melting peak was found during the dissociation protocol. Additionally, PCR products were run on a 1.2% agarose gel containing GelRed™ (Biotium). After electrophoresis only a single band was observed, which was cloned (TOPO^®^ TA cloning kit for sequencing, Invitrogen) and sequenced (Sanger sequencing, LGC Genomics, Berlin, Germany) to confirm target specificity. All q-RT-PCR results were normalized to the transcript levels of the selected reference genes and calculated relative to the transcript level in a calibrator sample according to the comparative Ct method [67]. GraphPad Prism 5 (GraphPad Software, San Diego, CA, USA) was used to test the statistical significance of differences in gene expression levels.

#### 2.8.4. q-RT-PCR Temporal Profiling of JH Pathway Components

For the temporal profiling of *Scg*-Met, *Scg*-Tai, *Scg*-Krh1, *Scg*-JHAMT and *Scg*-CYP15, q-RT-PCR analyses were performed on a collection of RNA samples derived from adult male *S. gregaria* tissues. The investigated tissues were the fat body, the gonads, the accessory glands and the CA/CC collected from male locusts at different timepoints during their adult development. Tissue samples were stored at −80 °C and consisted of four pooled samples, each derived from five animals, for every tissue and time point. For these q-RT-PCR analyses, *Scg*-Act and *Scg*-Ef1a were selected by the geNorm software (Biogazelle, Zwijnaarde, Belgium) as having the most stable expression and were used as reference genes.

## 3. Results

### 3.1. Identification of Scg-Met and Scg-Tai cDNAs in S. gregaria

We identified partial cDNAs coding for one Met isoform (*Scg-Met*) (Genbank: MK855050) and one Tai isoform (*Scg*-*Tai*) (Genbank: MK442071) by BLAST searches in in-house *S. gregaria* transcriptome databases. Both sequences, verified by cloning and sequencing, were uploaded to the NCBI GenBank database (Figures S9 and S10). Based on the recently published genome data for *S. gregaria* [60], we were able to further extend the predicted amino acid sequences of both *Scg*-Met and *Scg*-Tai (Figures S1 and S4). Multiple sequence alignments of *Scg*-Met, as well as *Scg*-Tai, with selected orthologs from different species (NCBI) revealed very high similarities in protein sequence between *S. gregaria* and *L. migratoria* (84.5% and 87.1% for Met and Tai, respectively) (Figures S2 and S5; phylogenetic trees in Figures S3 and S6). The overall sequence similarities of *Scg*-Met and *Scg*-Tai with their orthologs in the other insect species included in this analysis appeared to be far lower (in the 13–28% and 15–53% range, respectively). Nevertheless, three conserved domains were identified in all these sequences: a basic helix-loop-helix domain (bHLH), a Pern-Arnt-Sim (PAS) domain and a PAS-11 domain. The bHLH domain in Tai orthologs was more specifically identified as a bHLH-PAS domain belonging to the steroid receptor coactivator (SRC) protein family.

### 3.2. Transcript Levels of JH Pathway Components in Untreated Adult Male Locusts

We first analyzed the expression profiles of key mediators of the JH pathway in several relevant tissues at physiologically relevant time points during adult development of male desert locusts (Figure S7). The transcript levels of *Scg-Met*, *Scg-Tai, Scg-Krh1*, the transcription factor acting downstream of the JH receptor complex, were analyzed in the fat body, the testes, the AG and the retrocerebral *corpora allata/corpora cardiaca* (CA/CC) complex of male adult locusts on day 3, day 7 and day 15 after their final molt. In addition, transcript levels of *Scg*-JHAMT and *Scg*-CYP15A1, the enzymes catalyzing the final steps in the JH biosynthetic pathway of the desert locust, were analyzed in the CA/CC at the same time points after the final molt. Within this timeframe, the male reproductive system fully develops, and the adult desert locusts gradually become sexually mature.

Transcript levels of *Scg-Met*, *Scg-Tai* and *Scg-Krh1* significantly increased from day 3, over day 7, to day 15 in the fat body of male desert locusts in the adult life stage (Figure S7). However, no significant changes in the levels of these transcripts were detected in either the testes or the AG.

In the CA/CC complex of male desert locusts, the relative transcript levels of *Scg*-*JHAMT* and *Scg-CYP15A1* significantly increased from day 3 to day 7 of the adult stage, and the relative transcript levels of *Scg*-*JHAMT* also significantly increased from day 7 to day 15 (Figure S7). Remarkably, in these CA/CC complexes the transcript levels of *Scg-Met* and *Scg-Krh1* were significantly lower on days 7 and 15, when compared to day 3.

### 3.3. Expression of MEKRE93 and JH Biosynthesis Genes in dsScg-Met Injected Males

**Figure 1:**
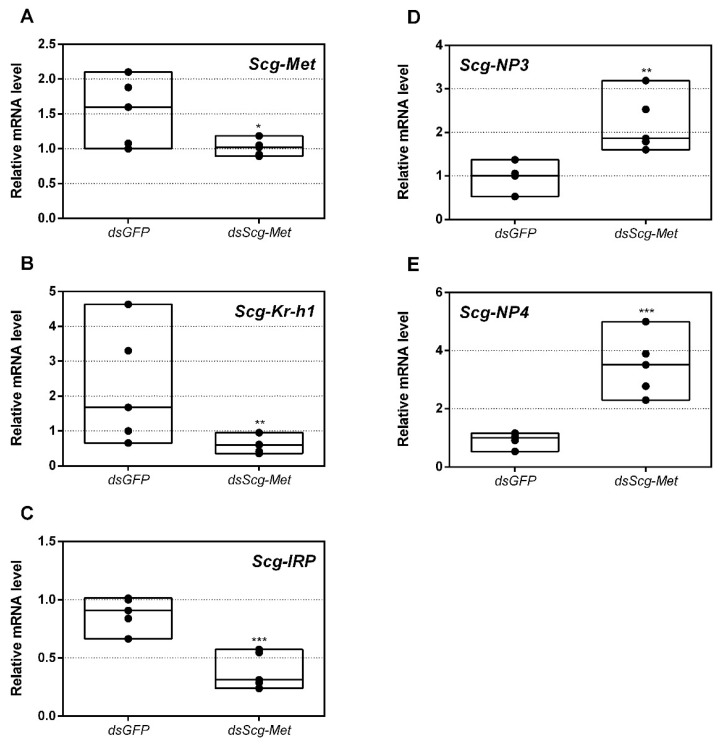
Relative mRNA levels measured in fat body tissue of *dsGFP* and *dsScg-Met* injected adult male desert locusts 34 days after the adult molt. (**A**) Transcript levels of *Scg-Met* were significantly reduced in the fat body demonstrating that the RNAi mediated knockdown was successful. Transcript levels of (**B**) *Scg-Krh1* and (**C**) *Scg*-IRP were significantly reduced, while transcript levels of (**D**) *Scg*-NP3 and (**E**) *Scg-NP4* were significantly increased in the fat body upon *dsScg-Met* treatment. Transcript levels in the fat body were normalized against two reference genes, *Scg-Act* and *Scg-Ef1a*. For every condition five biological replicates, each consisting of the pooled fat body samples from four individual locusts, were analyzed by q-RT-PCR. Data points are represented in a column graph as a floating bar (min to max value) with a line indicating the median. Significant differences (Student’s T-test) are indicated with asterisks (* *p* < 0.05; ** *p* < 0.01; *** *p* < 0.001). The following statistical p-values were obtained: *Scg-Met* (*p* = 0.048), *Scg-Krh1* (*p* = 0.0021), *Scg-IRP* (*p* = 0.0008), *Scg-NP3* (*p* = 0.0059), *Scg-NP4* (*p* = 0.0007). (Abbreviations: ds = double stranded, *Scg* = *Schistocerca gregaria*, GFP = Green Fluorescent Protein, Met = Methoprene-tolerant, Kr-h1 = Krüppel-homolog 1, IRP = insulin-related peptide, NP = neuroparsin, Act = actin, Ef1a = elongation factor 1-alpha).

**Figure 2:**
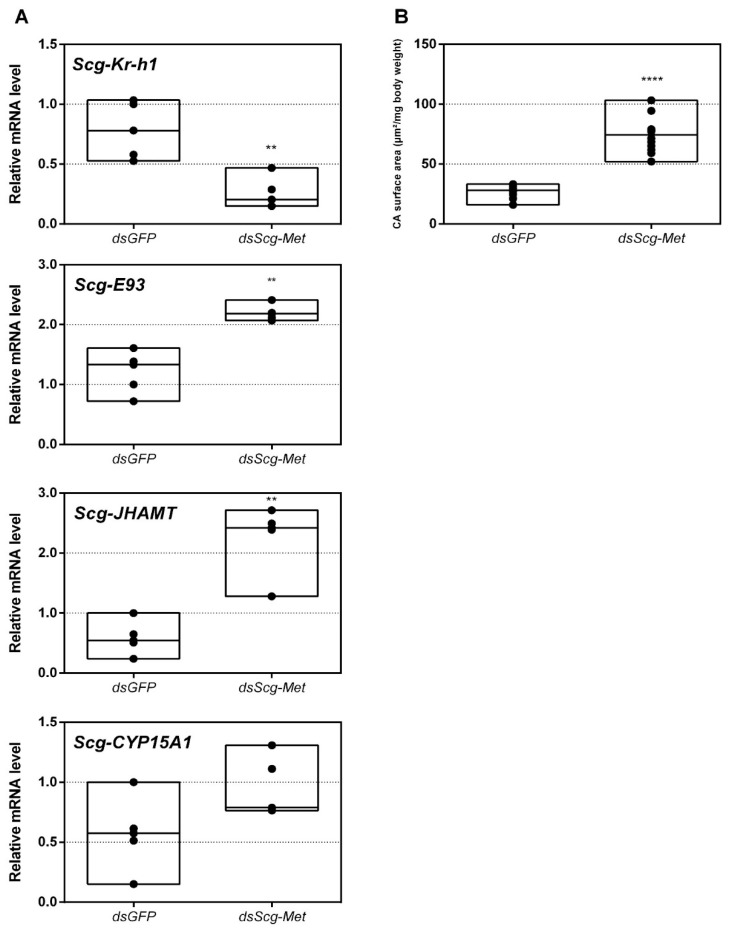
(**A**) Relative mRNA levels of *Scg-Krh1, Scg-E93, Scg-JHAMT* and *Scg-CYP15A* measured in the *corpora allata* (CA) of *dsGFP* and *dsScg-Met* injected adult male desert locusts 34 days after the adult molt. Data highlight the reduced *Scg-Krh1* transcript levels, as well as the increased *Scg-E93* and *Scg-JHAMT* transcript levels, in the CA after *Scg-Met* silencing. No significant change in the transcript levels of *Scg-CYP15A1* was detected. Transcript levels in the CA were normalized against two reference genes, *Scg*-*Act* and *Scg*-*Ef1a*. Data points are represented in a column graph as a floating bar (min to max value) with a line indicating the median. For every condition five biological replicates, each consisting of the pooled CA samples from four individual locusts, were analyzed by q-RT-PCR. Significant differences (Mann-Whitney U-test) are indicated with asterisks (** *p* < 0.01; **** *p* < 0.0001). The following statistical p-values were obtained: *Scg-Kr-h1* (*p* = 0.0079), *Scg-E93* (*p* = 0.0079), *Scg-JHAMT* (*p* = 0.008), *Scg-CYP15A1* (*p* = 0.06). (**B**) Normalized surface area of the CA in µm^2^/mg body weight of *dsGFP* and *dsScg-Met* treated animals. These data demonstrate that the CA significantly increased in size after *dsScg-Met* treatment. Data points are represented in a column graph as a floating bar (min to max value) with a line indicating the median. For each condition 13 animals were dissected for their CA. Significant differences (Student’s T-test) are indicated with asterisks (**** *p* < 0.0001). (Abbreviations: ds = double stranded, *Scg* = *Schistocerca gregaria*, GFP = Green Fluorescent Protein, Met = Methoprene-tolerant, Kr-h1 = Krüppel-homolog 1, JHAMT = JH acid methyltransferase, CA = *corpora allata*, Act = actin, Ef1a = elongation factor 1-alpha).

The efficiency of the RNAi-mediated knockdown of *Scg-Met* was investigated by comparing the transcript levels of *Scg-Met* in the fat body of *dsScg-Met* and *dsGFP* treated locusts on the day of dissections, 34 days after the adult molt. Our data indeed show a significant reduction of *Scg-Met* transcript levels, by an average of 44%, in the fat body of *dsScg-Met* injected locusts (*p* = 0.048) (Figure 1A). Moreover, the mRNA levels of the downstream factor *Scg*-Krh1, generally considered as an important indicator of the Met-mediated JH signaling activity, were significantly reduced in both the fat body (p = 0.0021) and the CA (*p* = 0.0079) of *dsScg-Met* injected adult males, when compared to the *dsGFP* injected control animals (Figure 1B and Figure 2A). On the other hand, we detected a significant increase of the mRNA levels of the transcription factor *Scg*-E93 (*p* = 0.0079) and of the JH biosynthetic enzyme *Scg*-JHAMT (*p* = 0.008) in the CA of *dsScg-Met* injected males compared to control (*dsGFP*) locusts, while an upward (but non-significant) trend was observed for the *Scg-CYP15A1* transcript levels (*p* = 0.06) (Figure 2A).

### 3.4. Absence of Sexual Activity in dsScg-Met Injected Male Locusts

Since day 12 after the adult molt, all *dsGFP* injected (control) males demonstrated normal copulation behavior. However, during the entire timeframe of the experiment (34 days), none of the *dsScg-Met* injected (experimentally treated) animals had displayed any sexual activity. In both *dsScg-Met* as well as *dsGFP* group, a total of 25 animals was observed.

### 3.5. Scg-Met Knockdown Increases Corpora Allata Size

Upon dissection, 34 days after the adult molt, we observed that the CA of *dsScg-Met* injected animals were larger than the CA of control animals (*p* < 0.0001) (Figure 2B). On average the normalized surface area of the CA in control animals was 26.83 µm^2^/mg body weight, while the surface of the normalized surface area of the CA in *dsScg-Met* treated animals was 73.98 µm^2^/mg body weight.

### 3.6. Scg-Met Knockdown Reduces Yellow Pigmentation in The Cuticle of Adult Male Desert Locusts

**Figure 3:**
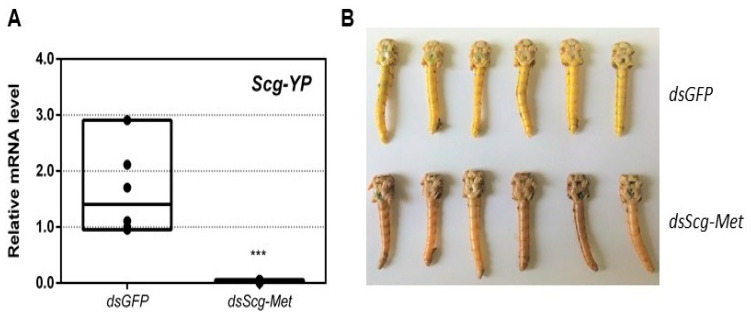
(**A**) Relative mRNA levels of *Scg-YP* measured in the epidermis of *dsGFP* and *dsScg-Met* injected adult male desert locusts 34 days after the adult molt. *Scg-YP* transcript levels were significantly lower after Met silencing. Transcript levels in the epidermis were normalized against two reference genes, *Scg-Act* and *Scg-Ef1a*. Data points are represented in a column graph as a floating bar (min to max value) with a line indicating the median. For every condition six biological replicates, each consisting of the pooled epidermis samples from three individual locusts, were analyzed by q-RT-PCR. Significant differences (Student’s T-test) are indicated with asterisks (*** *p* < 0.001) (the statistical p-value obtained for *Scg-YP* was 0.0002). (**B**) Pictures of the abdomen of both *dsGFP* treated (top, yellow) and *dsScg-Met* treated (bottom, beige/brown) male locusts 34 days after the adult molt. In clear contrast with the *dsScg-Met* injected locusts, all control animals displayed normal copulation behavior and had the bright yellow cuticular coloration that is typical for sexually mature gregarious desert locust males. (Abbreviations: ds = double stranded, *Scg* = *Schistocerca gregaria*, GFP = Green Fluorescent Protein, Met = Methoprene-tolerant, YP = yellow protein, Act = actin, Ef1a = elongation factor 1-alpha).

While all control animals turned bright yellow, knockdown of *Scg*-Met resulted in the absence of this yellow pigmentation (Figure 3B). Upon dissection, 34 days after the adult molt, the relative quantity of *Scg*-*YP* transcript, which codes for the carotenoid-binding Yellow Protein [68], was significantly lower in the epidermis of *dsScg-Met* injected males, when compared to the *dsGFP* injected ones (*p* = 0.0002) (Figure 3A).

### 3.7. Effect of Scg-Met Knockdown on Phenylacetonitrile Production

**Figure 4:**
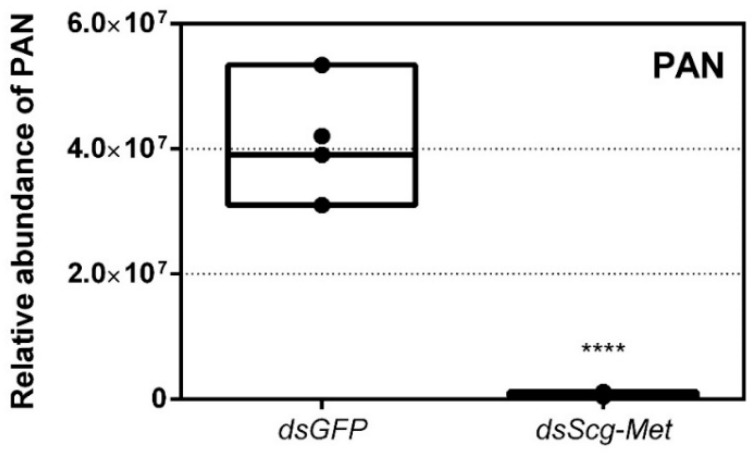
Relative abundance of PAN released by *dsGFP* and *dsScg-Met* injected adult male desert locusts 26 days after the adult molt. The emission of PAN was significantly reduced when *Scg-Met* was silenced. Data points are represented in a column graph as a floating bar (min to max value) with a line indicating the median. Significant differences (Student’s T-test) are indicated with asterisks (**** *p* < 0.0001). For each condition five individual animals were analyzed. PAN emission was measured by gas chromatography–mass spectrometry (GC–MS). (Abbreviations: ds = double stranded, *Scg* = *Schistocerca gregaria*, GFP = Green Fluorescent Protein, Met = Methoprene-tolerant, PAN = phenylacetonitrile).

On day 26 of the adult stage, gas chromatography–mass spectrometry (GC–MS) was used to compare the PAN pheromone emission between *dsGFP* (control group) and *dsScg-Met* injected (experimental group) animals. These measurements demonstrated a very drastic reduction of PAN emission when *Scg-Met* was knocked down (*p* < 0.0001) (Figure 4). In contrast, the control animals showed normal PAN emission spectra.

### 3.8. Effect of Scg-Met Knockdown on Development of Male Reproductive Organs

**Figure 5:**
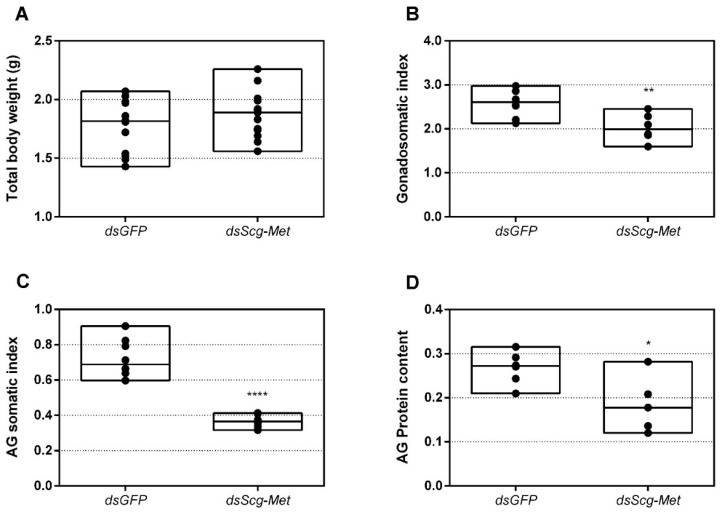
(**A**) Total body weight in grams of *dsGFP* and *dsScg-Met* injected adult male desert locusts 34 days after the adult molt. No significant differences (Student’s T-test) in body weight were observed between both conditions (the statistical p-value obtained for total body weight was 0.2414). For each condition 12 animals were analyzed. (**B**) Gonadosomatic index (GSI) of *dsGFP* and *dsScg-Met* injected adult male desert locusts 34 days after the adult molt. The GSI was significantly lower in *dsScg-Met* locusts compared to control animals. This result indicates the impaired development of the testes upon silencing of *Scg*-Met. Significant differences (Student’s T-test) are indicated with asterisks (** *p* < 0.01) (the statistical p-value obtained for GSI was 0.0014). For each condition 8 animals were analyzed. (**C**) Accessory gland somatic index (AGSI) of *dsGFP* and *dsScg-Met* injected adult male desert locusts 34 days after the adult molt. The AGSI was significantly lower in *dsScg-Met* injected locusts compared to *dsGFP* (control) animals. This result indicates the impaired development of the accessory glands (AG) upon silencing of *Scg*-Met. Significant differences (Student’s T-test) are indicated with asterisks (**** *p* < 0.0001). For each condition 8 animals were analyzed. (**D**) Normalized protein content of the accessory glands of *dsGFP* and *dsScg-Met* injected adult male desert locusts 34 days after the adult molt. The protein content of the AG was determined using the bicinchoninic acid assay (BCA) method and normalized to the respective AG weight. The relative protein content in the AG of *dsScg-Met* injected animals was significantly reduced compared to that of *dsGFP* injected (control) locusts. Significant differences (Student’s T-test) are indicated with asterisks (* *p* < 0.05) (the statistical p-value obtained for relative protein content in AG was 0.0255). For each condition 6 animals were analyzed. For all graphs, data points are represented in a column graph as a floating bar (min to max value) with a line indicating the median. (Abbreviations: ds = double stranded, *Scg* = *Schistocerca gregaria*, GFP = Green Fluorescent Protein, Met = Methoprene-tolerant).

We also examined the effect of *Scg-Met* knockdown on the weights of the testes and AG in adult male locusts. Thirty-four days after the first injections, the total body weight, as well as the weights of the testes and AG of both the *dsGFP* and the *dsScg-Met* treated animals were determined for the calculation of the GSI and AGSI, respectively (Figure 5A–C). At this point, none of the *dsScg-Met* injected males had initiated copulation with their female conspecifics, while on the other hand all control animals were sexually active. In line with this observation, animals subjected to a *Scg-Met* knockdown had significantly lower GSI (*p* = 0.0014) and AGSI (*p* < 0.0001) indices, when compared to the control animals (Figure 5B–C). Lower GSI and AGSI indicate lower normalized weights of both testes and accessory glands in the *Scg-Met* knockdown animals than in the controls. The impaired male reproductive organ development is further evidenced by the significantly lower normalized protein content of the AG in *dsScg-Met* injected locusts, when compared to the *dsGFP* control animals (*p* = 0.0255) (Figure 5D).

### 3.9. Effect of Scg-Met Knockdown on Gene Expression Profiles in the Fat Body

In this study, we also determined the levels of three developmental peptide hormone encoding transcripts upon *dsGFP* or *dsScg-Met* injection. Thirty-four days after the adult molt, we observed significantly elevated *Scg*-*NP3* (*p* = 0.0059) and *Scg*-*NP4* (*p* = 0.0007) and downregulated *Scg*-*IRP* (*p* = 0.0008) transcript levels in the fat body of *dsScg-Met* treated animals, when compared to the *dsGFP* injected control animals (Figure 1C–E). Interestingly, upon dissection, we also observed that the fat body in the *dsScg-Met* treated animals was more pronounced than in the *dsGFP* controls (Figure S8).

### 3.10. Knockdown of Scg-Tai also Results in an Identical Phenotype

**Figure 6:**
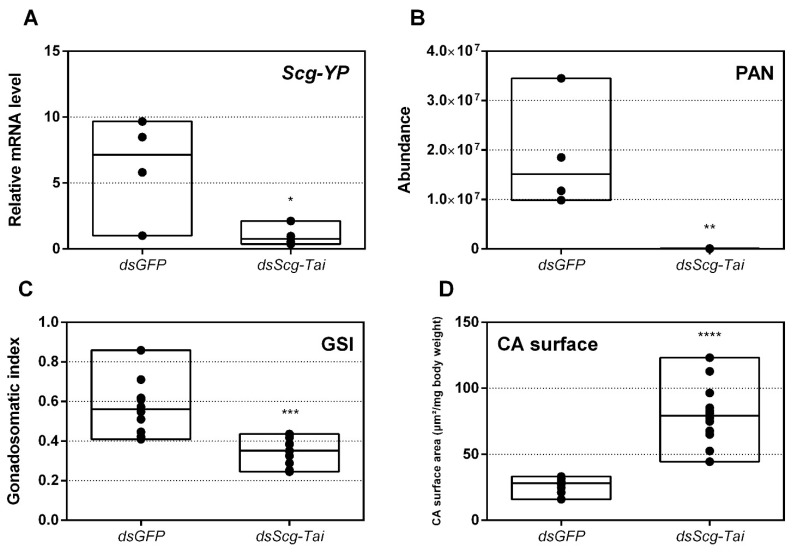
(**A**) Relative mRNA levels of *Scg-YP* measured in the epidermis of *dsGFP* and *dsScg-Tai* injected adult male desert locusts 34 days after the adult molt. *Scg-YP* transcript levels were significantly reduced after *Scg-Tai* silencing. Transcript levels in the epidermis were normalized against two reference genes, *Scg-Act* and *Scg-Ef1a*. For every condition four biological replicates, each consisting of epidermis samples from four individual locusts, were analyzed. Significant differences (Student’s T-test) are indicated with asterisks (* *p* < 0.05) (the statistical p-value obtained for *Scg-YP* was 0.0371). (**B**) Relative abundance of PAN released by *dsGFP* (control) and *dsScg-Tai* injected adult male desert locusts 26 days after the adult molt. The emission of PAN was significantly reduced when *Scg-Tai* was silenced. PAN emission was measured by gas chromatography–mass spectrometry (GC–MS). For every condition four individual animals were analyzed. Significant differences (Student’s T-test) are indicated with asterisks (** *p* < 0.01) (the statistical p-value obtained for PAN emission was 0.0018). (**C**) Gonadosomatic index (GSI) of control animals compared to *dsScg-Tai* treated animals. The GSI is significantly lower in *dsScg-Tai* treated animals compared to the control animals. This result indicates the impaired development of the testes upon silencing of *Scg-Tai*. For each condition 12 animals were analyzed. Significant differences (Student’s T-test) are indicated with asterisks (*** *p* < 0.001) (the statistical *p*-value obtained for GSI was 0.0001). (**D**) Normalized surface area of the CA in µm^2^/mg body weight of *dsGFP* and *dsScg-Tai* treated animals. These data demonstrate that the CA significantly increased after *dsScg-Tai* treatment. For each condition 12 animals were analyzed. Significant differences (Student’s T-test) are indicated with asterisks (**** *p* < 0.0001). For all graphs, data points are represented in a column graph as a floating bar (min to max value) with a line indicating the median. (Abbreviations: ds = double stranded, *Scg* = *Schistocerca gregaria*, GFP = Green Fluorescent Protein, Tai = Taiman, YP = yellow protein, Act = actin, Ef1a = elongation factor 1-alpha).

Earlier research in *Aedes aegypti*, *T. castaneum* and *L. migratoria* described the interaction of JH-bound Met with Tai to become a functional JH receptor complex that mediates downstream JH signaling via Kr-h1 [11,12]. Therefore, we also decided to perform an RNAi mediated knockdown of *Scg-Tai* in a similar way as described for the *Scg-Met* knockdown experiment. Animals were treated in a similar way as during the *Scg-Met* knockdown experiment and were also sacrificed at day 34 after molting to the adult stage. Similarly, as observed upon knockdown of *Scg-Met*, the *dsScg-Tai* injected males lacked yellow pigmentation in their cuticle and, in line with this, their *Scg-YP* transcript level was significantly reduced compared to *dsGFP* injected control animals (*p* = 0.037) (Figure 6A). In addition, they also showed a drastically reduced PAN pheromone emission (*p* = 0.0018) (Figure 6B). Furthermore, the *dsScg-Tai* injected animals had a significantly reduced GSI (*p* = 0.0001) (Figure 6C). Accordingly, none of the *Scg-Tai* knockdown males demonstrated copulation behavior, as opposed to all *dsGFP* injected control males. In both *dsGFP* as well as *dsScg-Tai* treatment group, a total of 20 animals was observed. Moreover, similarly as described above for the *dsScg-Met* condition, we observed a significant increase in the normalized surface areas of the CA after *dsScg-Tai* treatment in comparison with the controls (*p* < 0.0001) (Figure 6D). On average, the normalized surface area of the CA in control animals was 26.83 µm^2^/mg body weight, while the normalized surface area of the CA in *dsScg-Tai* treated animals was 80.3 µm^2^/mg body weight. Additionally, the fat body of the *dsScg-Tai* injected males was more prominent than in locusts of the control condition and had a similar appearance as observed in the *dsScg-Met* injected condition (Figure S8).

## 4. Discussion

The body of research focusing on the role of the JH receptor components, Met and Tai, in sexual maturation of male insects is out of balance with the vast amount of information available for female insects. In females of several insect species, a stimulatory role of JH on vitellogenin production by the fat body and on oocyte development in the ovary is evidenced [48]. Additionally in adult *S. gregaria* females, we recently described that the JH receptor Met is necessary for ovarian maturation, vitellogenesis and associated ecdysteroid biosynthesis, making it a crucial gene in female locust reproductive physiology [14]. In the current study, we report on the effects of an RNAi-mediated knockdown of *Scg*-*Met* and *Scg-Tai* on the reproductive development of adult male desert locusts. Our observations from these RNAi experiments indicate that in male *S. gregaria* both JH receptor components are essential for sexual maturation.

### 4.1. Scg-Met Knockdown Severely Disrupts the Reproductive Development of Adult Male Locusts

*Scg-Met* mRNA levels were significantly reduced by injecting the locusts with *dsScg-Met* indicating that the RNAi procedure was effective (Figure 1A). In addition, we also investigated the levels of *Scg-Krh1* and *Scg-E93*, situated downstream of Met in the MEKRE93 pathway in juvenile insects. The significant reduction in *Scg-Krh1* demonstrates that JH signaling was impaired upon knocking down *Scg*-Met (Figure 2A). E93 is an ecdysone inducible transcription factor, of which the expression is inhibited by Kr-h1, and vice versa [23,69,70,71,72]. In line with this, an increase in the relative mRNA levels of *Scg*-E93 was observed in the CA (Figure 2A). Therefore, the contrasting expression profiles of *Scg-Krh1* and Scg-*E93* are very well in agreement with the sustained *Scg-Met* knockdown that was obtained by the regular *dsScg-Met* injections, suggesting that a mutually inhibiting cross-talk between *Scg*-Kr-h1 and *Scg*-E93 also persists in adult male locusts.

Knockdown of *Scg-Met* resulted in very strong phenotypic effects, indicating a pivotal role of this gene in the life history of male desert locusts:

1. An obvious visual effect of the *Scg-Met* knockdown was the impaired cuticular pigmentation of the adult males (Figure 3). *S. gregaria* adults are weakly pigmented after their final molt. Hardening of the cuticle upon molting, results in steady background coloration. In adult gregarious males, a remarkable change to a bright yellow cuticular coloration occurs when they become sexually mature [73]. In our colony, this color change normally manifests itself at days 9–12 after the final molt. Research has highlighted the role of yellow protein (YP) in this remarkable phenotypic transformation. YP has a high binding capacity for carotenoids originating from the diet and will be allocated towards the epidermis and cuticle when gregarious males become sexually mature. High levels of JH during adult male development will induce increasing YP levels, eventually resulting in the deposition of the yellow pigment in the cuticle [74]. The strongly reduced *Scg-YP* expression in *dsScg-Met* injected males is fully in line with the observed absence of a bright yellow coloration in the cuticle, in stark contrast with the *dsGFP* injected (control) males (Figure 3).
2. In comparison to *dsGFP* injected (control) males, the *Scg-Met* knockdown locusts produced much lower amounts of the pheromone PAN (Figure 4). Gregarious desert locusts live in very dense populations. Besides visual cues, pheromone communication plays a crucial role in mating behavior. One of the best-known pheromones produced by gregarious male desert locusts is phenylacetonitrile (PAN). This pheromone is abundantly released by the legs and wings of gregarious male locusts and plays a crucial role in identifying an appropriate sexual partner by preventing male-male interactions, as well as in the post-copulatory guarding of the inseminated female mate [38]. Knockdown of *Scg-Met* very strongly affected PAN emission, which illustrates that *Scg*-Met plays a crucial role in the emission of this male-to-male anti-aphrodisiac pheromone.
3. The development of the male reproductive organs, testes and accessory glands, was significantly inhibited by knocking down *Scg-Met* during the adult stage (Figure 5). Interestingly, a recent study demonstrated that treating male *S. gregaria* nymphs (final nymphal stage) with a JH mimic resulted in underdeveloped accessory glands and seminal vesicles, and impaired molting to the adult stage [75]. This suggests a different function of JH in the development of reproductive organs in the desert locust during distinct life stages, again highlighting the complex role of JH throughout the life history of insects. The role of JH in protein accumulation in AG has been described in several insect species [76]. A more recent study on *D. melanogaster Met^2^*^7^ mutants first demonstrated the role of Met in the development of the male AG. *Met^2^*^7^ mutants showed reduced AG sizes. This could be ascribed to the overall reduced protein synthesis resulting from the impaired JH signaling [77]. Similar effects were observed after knocking down *Met* or *Tai* in the linden bug, *Pyrrhocoris apterus* [78,79]. These studies demonstrated the participation of Met and Tai in generating the JH dependent effects on AG size in male *P. apterus*. Surprisingly, Met knockdown in this species did not reduce locomotor activity or mating behavior, in contrast to what is observed during adult reproductive diapause, which made the authors suggest that an additional photoperiodic control mechanism might be situated in parallel, independent or upstream of JH [79]. In the red flour beetle, *T. castaneum,* a JHAMT knockdown reduced the AG size and production of seminal proteins, significantly affecting male fitness [80]. Moreover, in locust species, JH has previously been shown to be essential for the growth of the AG and associated protein content in this tissue [44,46,47]. Our results show that *Scg-Met* knockdown in *S. gregaria* produced effects opposite to JH itself, clearly illustrating the adverse consequences of a disabled transduction of the JH signal on the development of reproductive organs in adult male locusts.
4. Interestingly, both the size of the CA and the expression of *Scg-JHAMT*, which codes for a rate-limiting JH biosynthetic enzyme, were significantly increased upon sustained knockdown of *Scg-Met* (Figure 2). These observations suggest the existence of homeostatic regulatory mechanisms for preserving a balance between JH synthesis and JH sensitivity. This is also supported by the observed increase in *Scg-JHAMT* and *Scg-CYP15A1* expression upon *Scg-Met* knockdown in adult female *S. gregaria* [14]. A similar stimulation of JH production following Met knockdown was previously also described in the linden bug, *P. apterus* [78]. This homeostatic regulation may reside within the CA but could also be more complex and include inter-organ communication mechanisms.
5. Finally, knockdown of either component of the JH receptor complex induced very similar phenotypic effects in the desert locust (Figure 6). Recent studies revealed that Met forms a complex with Tai to become a functionally active JH-dependent receptor. Once JH is bound to Met, the JH-Met complex will subsequently interact with a Tai transcription factor domain to form an active JH signal transducer [10]. Our findings are in line with this mechanistic model for JH signaling and indicate that reproductive organ growth, yellow coloration and PAN pheromone emission in gregarious males are highly dependent on expression of both *Scg-Met* and *Scg-Tai*.

### 4.2. Profiles of Scg-Met, Scg-Tai, Scg-Krh1, Scg-JHAMT and Scg-CYP15A1 in Untreated Adult Males

In this study, we have also analyzed the transcript profiles of *Scg-Met*, its dimerization partner *Scg-Tai* and the downstream factor *Scg-Krh1* during male adult development in four different tissues and on three different time points after the final molt (Figure S7). The abundance of the transcripts coding for these JH pathway components significantly increased in the fat body during male reproductive maturation, showing lowest levels when males were sexually immature (day 3), intermediate levels when they were maturing (day 7) and highest when they had reached sexual maturity (day 15) (Figure S7). On day 15, males in our gregarious desert locust colony have fully developed gonads, have a bright yellow colored cuticle and are sexually active as manifested by their mating behavior. That the levels of all three transcripts significantly increased from day 3, over day 7 to day 15 in male fat body indicates that JH directly interacts with male fat body. This increased expression of receptor components in the fat body seems to correlate with the significant rise of mRNA levels for the JH biosynthetic enzymes *Scg*-JHAMT and *Scg*-CYP15A1 in the CA, both crucial for JH biosynthesis. This is also in line with the observed increase in *Scg-Krh1* expression, which is known to be activated downstream of the interaction of JH with Met and of JH-bound Met with Tai. These data may point at a prominent role of the fat body in generating JH-dependent responses that alter the locust’s metabolism in order to regulate the physiological development of male reproductive organs. Interestingly, in CA/CC complexes the transcript levels of *Scg-Met, Scg-Tai* and *Scg-Krh1* were significantly lower on days 7 and 15 when compared to day 3. Together with findings represented in Figure 2 and Figure 6, which show the significantly increased *Scg-JHAMT* expression, as well as larger CA size, upon *Scg-Met* or *Scg-Tai* knockdown, this observation strongly suggests the existence of homeostatic control mechanisms for inversely regulating JH biosynthesis and JH signaling activity within the CA.

### 4.3. Possible Cross-Talk with Developmental Peptide Hormones?

Considering the important role of the fat body in metabolic, developmental and reproductive regulation, we have investigated the levels of *Scg-IRP*, *Scg-NP3* and *Scg-NP4*, three important peptide hormone encoding transcripts that are known to be expressed in the fat body and play a role in female reproductive physiology [51,81]. When compared to *dsGFP* injected control males, the *dsScg-Met* injected ones had significantly reduced *Scg-IRP* levels in the fat body (Figure 1C). In many metazoans, the insulin/IGF signaling pathway (ISP) is an important sensor of the nutritional and metabolic status, as well as a regulator of anabolic processes that result in growth and/or reproduction [48,57]. In *S. gregaria,* only one insulin-related peptide (IRP) (*Scg*-IRP) with main expression in brain and fat body was identified ([56]. Previous studies revealed that *Scg*-IRP expression is regulated during the reproductive cycle and there is evidence for a stimulatory role of *Scg*-IRP on vitellogenesis and ovarian development in adult female desert locusts [51]. In several other insect species, complex functional interactions have been observed between the ISP and JH signaling [7,14,48,82]. In the fruit fly, *D. melanogaster*, the expression of the insulin receptor (IR) was detected in the CA, indicating the ability of the CA to respond to changing insulin-like peptide levels. Moreover, *D. melanogaster IR* mutants showed significantly reduced JH levels [83]. In the mosquito *Culex pipiens,* the administration of JH rescued the defects observed upon silencing of the ISP [84]. A microarray analysis to explore gene expression in response to the administration of a JH analog in the silkworm, *Bombyx mori,* demonstrated a stimulatory effect on key regulatory genes involved in the ISP [85]. In the red flour beetle, *T. castaneum,* JH signaling was shown to stimulate the expression of two insulin-like peptide encoding genes [86]. Moreover, in this species, silencing of the JH receptor Met resulted in decreased insulin-like peptide transcript levels [87]. Therefore, our data of the current study extend these widely observed links between JH signaling and ISP towards reproductive development of adult male desert locusts. In this process, an important role seems to be played by the fat body, where the expression of *Scg-Met*, *Scg-Tai* and *Scg-Krh1* significantly increased during male sexual maturation (Figure S7), and where in *dsScg-Met* injected males significantly reduced transcript levels were observed for both *Scg-Met* and *Scg-IRP* (Figure 1). In addition, the fat body appeared to be more pronounced in both *Scg-Met* and *Scg-Tai* knockdown males than in the *dsGFP* injected (control) ones (Figure S8). Interestingly, fat body hypertrophy is generally observed in insects with reduced JH and ISP signaling, and in several species this is a naturally occurring phenomenon when the animal is going into a reproductive diapause [48]. This happens in situations when the environmental conditions are adverse, and energy and nutrients are rather saved for the individual insect’s survival than invested in gonad development and reproduction. Moreover, it has been shown that a reproductive diapause can be artificially induced in the mosquito *C. pipiens* by knocking down the insulin receptor via RNAi [84]. Since there appears to exist a functional relationship between nutrient availability, ISP, JH signaling and reproductive maturation, an interesting question could be whether Met knockdown might also affect food intake. There are some studies reporting on a possible involvement of JH in the regulation of digestion by controlling the expression of digestive serine proteases in response to food uptake [88,89,90,91,92]. In 2007, Meunier et al. also reported that Takeout, a putative JH binding protein, is essential for nutritional homeostasis in the fruit fly, *Drosophila melanogaster*, by modulating circulating JH levels and creating a link between circadian rhythm and feeding behavior [93]. In our current study, all animals in the different conditions were fed daily in the same manner. Although we didn’t notice any obvious differences in food consumption, we haven’t systematically monitored individual food intake levels. Therefore, additional experimental analyses would be required to answer this question.

While the levels of *Scg-Met* and *Scg-IRP* mRNAs in the fat body of *dsScg-Met* injected males were reduced, we observed significantly elevated expression of *Scg*-*NP3* and *Scg*-*NP4*. In female locusts, the effects generated by NPs are opposite to these of JH and IRP [51], although the exact in vivo relationship with these pathways remains elusive. Four closely related members of the neuroparsin family have previously been cloned from *S. gregaria*, but mRNA levels of only two (*Scg-NP3* and *Scg-NP4*) are predominantly localized in the fat body of gregarious desert locusts [51,81,94]. In locusts, the levels of these neuroparsin transcripts show temporal changes during the hormonally controlled molting and reproductive cycles, while locust phase dependent differences in NP expression have also been reported [81,95]. Interestingly, in line with the observed sequence similarity of NPs with a highly conserved region in vertebrate IGBP, *Scg*-NP4 was shown capable of interacting in vitro with *Scg*-IRP [56]. When knocking down all four neuroparsins in female locusts, a clear increase was observed in vitellogenin transcript levels in the fat body and in basal oocyte size in the ovarian follicles ([51]. In the present study, we observed a remarkably opposite effect of *Scg-Met* knockdown on *Scg-NP3/4* expression in comparison to *Scg-IRP*. Interestingly, knockdown of *Scg-Met* also resulted in similar effects on *Scg-IRP* and *Scg-NP3/4* expression in adult female *S. gregaria* [14]. Increased NP and decreased IRP levels would be expected to strengthen the inhibitory effects on reproductive development imposed by knockdown of *Scg*-Met, the receptor responsible for JH signaling. Therefore, the changes in *Scg*-*IRP*, *Scg*-*NP3* and *Scg*-*NP4* expression observed after *Scg-Met* knockdown may have contributed to the arrested development of the male reproductive system, the inability to acquire mature gregarious male traits and the observed sexually inactive phenotype.

## 5. Conclusions

This study has demonstrated that the proper development of sexual traits in adult gregarious locust males can be inhibited in an early stage by injecting the locusts as of adult molting with either *dsScg-Met* or *dsScg-Tai*. The treatment resulted in individuals that showed no mating behavior, did not produce the associated pheromone and did not obtain the characteristic bright yellow color associated with sexually mature male locusts. Our results therefore indicate that these genes are crucial for the transduction of the JH signal in adult male *S. gregaria* and play a “master switch” role in sexual development. Our study clearly demonstrates the potential of manipulating locust male reproductive maturation as a means to control population growth. Specifically targeting the JH signaling system of locusts could therefore prove to be a very efficient and successful way by which swarming desert locust plagues can be prevented in a more biorational manner.

## Supplementary Materials

The following are available online at https://www.mdpi.com/2218-273X/11/2/244/s1, Figure S1: *Schistocerca gregaria* Methoprene-tolerant (*Scg*-Met) predicted protein sequence, Figure S2: Multiple sequence alignment of Met orthologs, Figure S3: Phylogenetic tree of Met orthologs from different insect species, Figure S4: *Schistocerca gregaria* Taiman (*Scg*-Tai) predicted protein sequence, Figure S5: Multiple sequence alignment of Tai orthologs, Figure S6: Phylogenetic tree of Taiman orthologs from different insect species, Figure S7: Relative mRNA levels of *Scg-Met*, *Scg-Tai* and *Scg-Krh1* measured in the fat body, gonads, accessory glands and CA/CC of male desert locusts at day 3, day 7 and day 15 following the adult molt, Figure S8: Dissected carcasses of *dsGFP* (control), *dsScg-Met* and *dsScg-Tai* injected adult male *Schistocerca gregaria*, Figure S9: *Schistocerca gregaria* Methoprene-tolerant (Met) mRNA, partial cds (MK855050), Figure S10: *Schistocerca gregaria* Taiman (Tai) mRNA, partial cds (MK442071). Table S1: Oligonucleotide sequences of primers used in dsRNA construct design, Table S2: Oligonucleotide sequences of primers used in q-RT-PCR, Table S3: List of species and accession numbers of protein sequences used in the phylogenetic analyses.
