# Supplementary material for: Crucial Role of Juvenile Hormone Receptor Components Methoprene-Tolerant and Taiman in Sexual Maturation of Adult Male Desert Locusts
Source: Biomolecules. 2021 Feb 9;11(2):244. doi: 10.3390/biom11020244 (PMC7915749; doi:10.3390/biom11020244)
Supplement: Supplementary file 1 [file biomolecules-11-00244-s001.zip › SupplementaryFiles/Supp_Figure_Captions-BIOM_revised.pdf]

Supplementary information

Figure S1. *Schistocerca gregaria* Methoprene-tolerant (*Scg*-Met) predicted protein sequence. Conserved protein domains are indicated: bHLH (blue), PAS domain (black) and PAS-11 domain (grey). PAS and PAS-11 domains are sometimes also designated as PAS-A and PAS-B, respectively.

Figure S2. Multiple sequence alignment of Met orthologs. Protein sequences were aligned using the Clustal Omega algorithm and visualized using Mview. Residues are colored by identity. The percentage of coverage (cov) and identity (pid) are shown with *Scg*-Met as a reference. The conserved protein domains are indicated: bHLH (blue bar), PAS (black bar), PAS-11 domain (grey bar). PAS and PAS-11 domains are sometimes also designated as PAS-A and PAS-B, respectively. (Abbreviations: Met = Methoprene-tolerant, GCE = germ cell expressed, Sg = *Schistocerca gregaria*, Lm = *Locusta migratoria*, Pk = *Planococcus kraunhiae*, Bg = *Blatella germanica*, Tc = *Tribolium castaneum*, Ha = *Helicoverpa armigera*, Dm = *Drosophila melanogaster*, Aa = *Aedes aegypti*, Dma = *Daphnia magna*).

Figure S3. Phylogenetic tree of Met orthologs from different insect species. The subsequent phylogenetic analysis was performed using the IQTREE web server and visualized using iTOL. The tree was rooted using Met from the crustacean *Daphnia magna* as an outgroup. The scale bar in the upper left corner represents evolutionary distance. Bootstrap values are shown at each node.

Figure S4. *Schistocerca gregaria* Taiman (*Scg*-Tai) predicted protein sequence. Conserved protein domains are indicated: bHLH (blue), PAS domain (black) and PAS-11 domain (grey). PAS and PAS-11 domains are sometimes also designated as PAS-A and PAS-B, respectively.

Figure S5. Multiple sequence alignment of Tai orthologs. Protein sequences were aligned using the Clustal Omega algorithm and visualized using Mview. Residues are colored by identity. The percentage of coverage (cov) and identity (pid) are shown with *Scg*-Tai as a reference.. The conserved protein domains are indicated: bHLH (blue bar), PAS (black bar), PAS-11 domain (grey bar). PAS and PAS-11 domains are sometimes also designated as PAS-A and PAS-B, respectively. (Abbreviations: Tai = Taiman, Sg = *Schistocerca gregaria*, Lm = *Locusta migratoria*, Pk = *Planococcus kraunhiae*, Bg = *Blatella germanica*, Dm = *Drosophila melanogaster*, Aa = *Aedes aegypti*, Mn = *Macrobrachium nipponense*).

Figure S6. Phylogenetic tree of Taiman orthologs from different insect species. The phylogenetic analysis was performed using the IQTREE web server and visualized using iTOL. The tree was rooted using Tai from the crustacean *Macrobrachium nipponense* as an outgroup. The scale bar in the upper left corner represents evolutionary distance. Bootstrap values are shown at each node.

Figure S7. Relative mRNA levels of *Scg-Met*, *Scg-Tai* and *Scg-Krh1* measured in the fat body, gonads, accessory glands and CA/CC of male desert locusts at day 3, day 7 and day 15 following the adult molt. Relative mRNA levels of *Scg-JHAMT* and *Scg-Cyp15* were only measured in the CA/CC complexes, since

the CA are the primary site of synthesis and activity for both enzymes. Transcript levels of *Scg-Met*, *Scg-Tai* and *Scg-Krh1* significantly increased from day 3, over day 7, to day 15 in the fat body of adult male desert locusts. However, no significant changes in the levels of these transcripts were detected in either the testes or the accessory glands. In the CA/CC complex of male desert locusts, the relative transcript levels of *Scg-JHAMT* and *Scg-CYP15A1* significantly increased from day 3 to day 7 of the adult stage, and the relative transcript levels of *Scg-JHAMT* also significantly increased from day 7 to day 15. In the CA/CC, the transcript levels of *Scg-Met* and *Scg-Krh1* were significantly lower on days 7 and 15, when compared to day 3. For every condition four biological replicates, each consisting of the pooled tissues from five individual locusts, were analyzed by q-RT-PCR. Transcript levels were normalized against two reference genes, *Scg-Act* and *Scg-Ef1a*. Data points are represented in a column graph as a floating bar (min to max value) with a line indicating the median. Significant differences (ANOVA) are indicated with asterisks (* $p < 0.05$; ** $p < 0.01$; *** $p < 0.001$; **** $p < 0.0001$). The following statistical p-values were obtained: for *Scg-Met* in the fat body: day 3 vs day 7 (p = 0.0036), day 3 vs day 15 (p = 0.0097), day 7 vs 15 (p = 0.9249); in the gonads: day 3 vs day 7 (p = 0.9997), day 3 vs day 15 (p = 0.4120), day 7 vs 15 (p = 0.3999); in the accessory glands: day 3 vs day 7 (p = 0.7108), day 3 vs day 15 (p = 0.7446), day 7 vs 15 (p = 0.9983); in the CA/CC: day 3 vs day 7 (p = 0.0249), day 3 vs day 15 (p = 0.0921), day 7 vs 15 (p = 0.9227). For *Scg-Tai* in the fat body: day 3 vs day 7 (p = 0.0009), day 3 vs day 15 (p = < 0.0001), day 7 vs 15 (p = 0.0003); in the gonads: day 3 vs day 7 (p = 0.8207), day 3 vs day 15 (p = 0.2850), day 7 vs 15 (p = 0.6191); in the accessory glands: day 3 vs day 7 (p = 0.9759), day 3 vs day 15 (p = 0.6211), day 7 vs 15 (p = 0.4922); in the CA/CC: day 3 vs day 7 (p = 0.4767), day 3 vs day 15 (p = 0.9253), day 7 vs 15 (p = 0.7588). For *Scg-Krh1* in the fat body: day 3 vs day 7 (p = < 0.0001), day 3 vs day 15 (p = < 0.0001), day 7 vs 15 (p = 0.0004); in the gonads: day 3 vs day 7 (p = 0.9098), day 3 vs day 15 (p = 0.7653), day 7 vs 15 (p = 0.9563); in the accessory glands: day 3 vs day 7 (p = 0.9967), day 3 vs day 15 (p = 0.9891), day 7 vs 15 (p = 0.9978); in the CA/CC: day 3 vs day 7 (p = 0.0473), day 3 vs day 15 (p = 0.0769), day 7 vs 15 (p = 0.9991), For *Scg-CYP15A1* in the CA/CC: day 3 vs day 7 (p = 0.0024), day 3 vs day 15 (p = 0.0018), day 7 vs 15 (p = 0.9560). For *Scg-JHAMT* in the CA/CC: day 3 vs day 7 (p = 0.0011), day 3 vs day 15 (p < 0.0001), day 7 vs 15 (p < 0.0001).

Figure S8. Dissected carcasses of *dsGFP* (control), *dsScg-Met* (dsMet) and *dsScg-Tai* (dsTai) injected adult male *Schistocerca gregaria*. These pictures clearly illustrate that the fat body in the *dsScg-Met* and *dsScg-Tai* injected males was more pronounced than in animals of the *dsGFP* injected control condition. The fat body is visible as the yellow-beige tissue in the carcasses of the dissected locusts, as indicated in the figure with red arrows.

Figure S9. *Schistocerca gregaria* Methoprene-tolerant (*Scg*-Met) encoding mRNA, partial cds (MK855050) with *dsScg-Met* primer sites (grey) and *Scg-Met* (q-)RT-PCR primer sites (black).

Figure S10. *Schistocerca gregaria* Taiman (*Scg*-Tai) encoding mRNA, partial cds (MK442071) with *dsScg-Tai* primer sites (grey) and *Scg-Tai* (q-)RT-PCR primer sites (black).
