# Supplementary material for: Crucial Role of Juvenile Hormone Receptor Components Methoprene-Tolerant and Taiman in Sexual Maturation of Adult Male Desert Locusts
Source: Biomolecules. 2021 Feb 9;11(2):244. doi: 10.3390/biom11020244 (PMC7915749; doi:10.3390/biom11020244)
Supplement: Supplementary file 1 [file biomolecules-11-00244-s001.zip › SupplementaryFiles/Supp_Table_BIOM_revised.docx]

**Table S1.** Oligonucleotide sequences of primers used in dsRNA construct design. T7 RNA polymerase promoter sequences necessary for efficient RNA transcription are underlined.

| **Target genes** | **Forward primer** | **Reverse primer** |
| --- | --- | --- |
| *Scg-Met* | 5’- TAATACGACTCACTATAGGGAGA AATGAGCCGTTTGGCAGTTCCA -3’ | 5’- TAATACGACTCACTATAGGGAGA GGCGGTGCAGCACTCATAGC -3’ |
| *Scg-Tai* | 5’- TAATACGACTCACTATAGGGAGA TCGGAGAATGTGGAGCAGTT-3’ | 5’-GAAATTAATACGACTCACTATAGGGCC AGGTCACCACTCTCAGAACG-3’ |
| *GFP* | 5’-TAATACGACTCACTATAGGGAGA AAGGTGATGCTACATACGGAA-3’ | 5’-TAATACGACTCACTATAGGGAGA ATCCCAGCAGCAGTTACAAAC-3’ |

**Abbreviations:** *Scg* = *Schistocerca gregaria*, Met = Methoprene-tolerant, Tai = Taiman, GFP = Green Fluorescent Protein.

**Table S2.** Oligonucleotide sequences of primers used in q-RT-PCR.

| **Reference genes** | **Forward primer** | **Reverse primer** |
| --- | --- | --- |
| *Scg-Act* | 5’-AATTACCATTGGTAACGAGCGATT-3’ | 5’-TGCTTCCATACCCAGGAATGA-3’ |
| *Scg-EF1α* | 5’-GATGCTCCAGGCCACAGAGA-3’ | 5’-TGCACAGTCGGCCTGTGAT-3’ |

| **Target genes** | **Forward primer** | **Reverse primer** |
| --- | --- | --- |
| *Scg-Met* | 5’-GGTGCCTGAAGAGGAAGAAA-3’ | 5’-ATGGAGGTGATGAAGGAGAAAG-3’ |
| *Scg-Tai* | 5’-GCCAGCTTTGCTGACATGAA-3’ | 5’-GGAGGATGGCGCACTTGT-3’ |
| *Scg-Krh1* | 5’-CTCCAAGACGTTCATCCAGAG-3’ | 5’-TGCTTGGAGCAGGTGAAG-3’ |
| *Scg-E93* | 5’-CGCAAGCAGACATAGAACCC-3’ | 5’-TGGTCTCAGGAACATCCCAC-3’ |
| *Scg-NP3* | 5’-GCGGAATCGGCATGGA-3’ | 5’-TCACAGAGCAACCGGAACATT-3’ |
| *Scg-NP4* | 5’-TGGCGACTCTCCAGTGCTT-3’ | 5’-TGACACATTCATTCCTCTTCTGACA-3’ |
| *Scg-IRP* | 5’-CCGTGGCAACTACAACACCAT-3’ | 5’-TCCGCGTCCGACACATCT-3’ |
| *Scg-JHAMT* | 5’-CGGAGCAAAGGCAAGCA-3’ | 5’-CCACTTCACCGCCTGGTTT-3’ |
| *Scg-Cyp15A1* | 5’-AAAGCAACTTCATCATTCACAGATG-3’ | 5’-CAGAGCCAGCCATGAACAAA-3’ |
| *Scg-YP* | 5’-ACGACAACACCACGGAAAAC-3’ | 5’-TGAAGGTGTCGCTATTGGTG-3’ |

**Abbreviations:** *Scg* = *Schistocerca gregaria*, Act = actin, Ef1a = elongation factor 1-alpha, Met = Methoprene-tolerant, Tai = Taiman, Krh1 = Krüppel-homolog 1, NP = neuroparsin, IRP = insulin-related peptide, JHAMT = JH acid O-methyltransferase, Cyp15A1 = methyl farnesoate epoxidase, YP = yellow protein.

**Table S3.** List of species and accession numbers of protein sequences used in the phylogenetic analyses.

| **Species** | **Accession number Met** | **Accession number Tai** |
| --- | --- | --- |
| *Schistocerca gregaria* | NA | NA |
| *Locusta migratoria* | AHA42531.1 | ANG56297.1 |
| *Planococcus kraunhiae* | BAU79435.1 | BAU79451.1 |
| *Blatella germanica* | CDO33887.1 | CDO33883.1 |
| *Drosophila melanogaster* | Met: NP_511126.2  GCE: AAF48439.2 | ADV36996.1 |
| *Aedes aegypti* | AAW82472.1 | AXN70147.1 |
| *Tribolium castaneum* | NP_001092812.1 | / |
| *Helicoverpa armigera* | AJW29006.1 |  |
| *Macrobrachium nipponense* | / | QLJ57681.1 |
| *Daphnia magna* | BAM83855.1 | / |
