# Supplementary material for: Crucial Role of Juvenile Hormone Receptor Components Methoprene-Tolerant and Taiman in Sexual Maturation of Adult Male Desert Locusts
Source: Biomolecules. 2021 Feb 9;11(2):244. doi: 10.3390/biom11020244 (PMC7915749; doi:10.3390/biom11020244)
Supplement: Supplementary file 1 [file biomolecules-11-00244-s001.zip › SupplementaryFiles/Supp_Table_BIOM_revised.pdf]

Table S1. Oligonucleotide sequences of primers used in dsRNA construct design. T7 RNA polymerase promoter sequences necessary for efficient RNA transcription are underlined.

Target genes	Forward primer	Reverse primer
Scg-Met	5’- TAATACGACTCACTATAGGGAGA<br>AATGAGCCGTTTGGCAGTTCCA -3’	5’- TAATACGACTCACTATAGGGAGA<br>GGCGGTGCAGCACTCATAGC -3’
Scg-Tai	5’- TAATACGACTCACTATAGGGAGA<br>TCGGAGAATGTGGAGCAGTT-3’	5’-GAAATTAATACGACTCACTATAGGGCC<br>AGGTCACCACTCTCAGAACG-3’
GFP	5’-TAATACGACTCACTATAGGGAGA<br>AAGGTGATGCTACATACGGAA-3’	5’-TAATACGACTCACTATAGGGAGA<br>ATCCCAGCAGCAGTTACAAAC-3’

Abbreviations: *Scg* = *Schistocerca gregaria*, Met = Methoprene-tolerant, Tai = Taiman, GFP = Green Fluorescent Protein.

Table S2. Oligonucleotide sequences of primers used in q-RT-PCR.

Reference genes	Forward primer	Reverse primer
Scg-Act	5’-AATTACCATTGGTAACGAGCGATT-3’	5’-TGCTTCCATACCCAGGAATGA-3’
Scg-EF1α	5’-GATGCTCCAGGCCACAGAGA-3’	5’-TGCACAGTCGGCCTGTGAT-3’

Target genes	Forward primer	Reverse primer
Scg-Met	5’-GGTGCCTGAAGAGGAAGAAA-3’	5’-ATGGAGGTGATGAAGGAGAAAG-3’
Scg-Tai	5’-GCCAGCTTTGCTGACATGAA-3’	5’-GGAGGATGGCGCACTTGT-3’
Scg-Krh1	5’-CTCCAAGACGTTCATCCAGAG-3’	5’-TGCTTGGAGCAGGTGAAG-3’
Scg-E93	5’-CGCAAGCAGACATAGAACCC-3’	5’-TGGTCTCAGGAACATCCCAC-3’
Scg-NP3	5’-GCGGAATCGGCATGGA-3’	5’-TCACAGAGCAACCGGAACATT-3’
Scg-NP4	5’-TGGCGACTCTCCAGTGCTT-3’	5’-TGACACATTCATTCCTCTTCTGACA-3’
Scg-IRP	5’-CCGTGGCAACTACAACACCAT-3’	5’-TCCGCGTCCGACACATCT-3’
Scg-JHAMT	5’-CGGAGCAAAGGCAAGCA-3’	5’-CCACTTCACCGCCTGGTTT-3’
Scg-Cyp15A1	5’-AAAGCAACTTCATCATTCACAGATG-3’	5’-CAGAGCCAGCCATGAACAAA-3’
Scg-YP	5’-ACGACAACACCACGGAAAAC-3’	5’-TGAAGGTGTCGCTATTGGTG-3’

Abbreviations: *Scg* = *Schistocerca gregaria*, Act = actin, Ef1a = elongation factor 1-alpha, Met = Methoprene-tolerant, Tai = Taiman, Krh1 = Krüppel-homolog 1, NP = neuroparsin, IRP = insulin-related peptide, JHAMT = JH acid O-methyltransferase, Cyp15A1 = methyl farnesoate epoxidase, YP = yellow protein.

Table S3. List of species and accession numbers of protein sequences used in the phylogenetic analyses.

Species	Accession number Met	Accession number Tai
Schistocerca gregaria	NA	NA
Locusta migratoria	AHA42531.1	ANG56297.1
Planococcus kraunhiae	BAU79435.1	BAU79451.1
Blatella germanica	CDO33887.1	CDO33883.1
Drosophila melanogaster	Met: NP_511126.2	ADV36996.1
	GCE: AAF48439.2	
Aedes aegypti	AAW82472.1	AXN70147.1
Tribolium castaneum	NP_001092812.1	/
Helicoverpa armigera	AJW29006.1	
Macrobrachium nipponense	/	QLJ57681.1
Daphnia magna	BAM83855.1	/
