# Supplementary material for: Crucial Role of Juvenile Hormone Receptor Components Methoprene-Tolerant and Taiman in Sexual Maturation of Adult Male Desert Locusts
Source: Biomolecules. 2021 Feb 9;11(2):244. doi: 10.3390/biom11020244 (PMC7915749; doi:10.3390/biom11020244)
Supplement: Supplementary file 1 [file biomolecules-11-00244-s001.zip › SupplementaryFiles/SuppFig1.pdf]

MRNKAEKQRRDKLNSLITELAHLVPMVAQSPRRMDKTSILRLSATFLRMYRCLPLKPGEIHEPFLPFLETLKVSQVLLDFL
QGMEGFMLVVTGAGKIVFVTHTVEKLLGHTQNDLLGQSLYNITRPEDHEELRRNIETAPSDGRASSAASGCSPALTSDES
SCEDGAAGGSTPSSSPSASRPGTSASTASVGSSCDGGRAGLGGLFGSAGAAEHHRRSFYIHLAQRAGGRAAGSGSATA
GAGGGGGGGGGAAGQGAEQTTYELVHFLGYLQVPRSHEPPPATHRPRRHRDTVPSSNSDNVLVAVVQLFREKKVME
LSLLEATLDEYQTRHLKDGSIVYSDHRISVVAGYLADEVHGHNAFTFMHQDDMAYALIALQQMFDKNEPFGSSTYRLVT
KNGQYIYMRTRGYLEFAHGSKDVETFLCINTLLSKEEGEDGLREMKQRFSAYVSALRAAGADLSLAGIAAAMSAAPPPPR
MVKGSELPKLPPPPPPPPPPSLQPSSPSPLPPPRPPPPPPPPQPPAAITSLSSPEIITNSTLCEVSPPFTAMVTESAASSEQSAD
VFGSVSRSEAAASKYRNQAPTAASRSPTVDPYLPAARPRAVSEGDDTTGFNLQNFVSLFKVPEEEENSVEVQLSPLANST
GITTDLEEKEQLRNPLSPSSPPSRHPYTAAQRIRAASESELQTANSGSTFIQSDPASRGGNWQFPRPGDMPHSGTTERRP
PERPTVITGRCCAGSERMKCSRLSVMSVTGDEKRITSVRGEVTVLKRTMSADVENPSTTKKLNVGPRYCRQRHRVEHPP
TEALSVEVNTLPPDELQHIILSRDSGMYNSQPTVLVSQHSLFRTSQPALQTVTQNVPASSLSPPQLAEMQSTSVTPPSFI
MEEPSGLPLVNLSDANTLSVGCQDPLISPDHGDSDSFSSLGLPDPENNPEVLDLLEASELKLNFDTLQEVRSSNEPEAPSG
PETAVTLERFLARKHLQLESCMQRQETQMHCIEHDLTQLPLADHVFRSNLTQLQASGPIHARKEERQASKLHIPVLTPIF
MRNNIRSTRYC
