# Supplementary material for: Crucial Role of Juvenile Hormone Receptor Components Methoprene-Tolerant and Taiman in Sexual Maturation of Adult Male Desert Locusts
Source: Biomolecules. 2021 Feb 9;11(2):244. doi: 10.3390/biom11020244 (PMC7915749; doi:10.3390/biom11020244)
Supplement: Supplementary file 1 [file biomolecules-11-00244-s001.zip › SupplementaryFiles/SuppFig10.pdf]

>MK442071.1 Schistocerca gregaria Tai protein mRNA, partial cds
ATGTCAACAGTCATCGCTGAAAATGCGGGGCTTGGCCCATGTGACCTCCAGGACCCTGTGTGGGTCAAGA
TGAGCGCTGCCGTCACTGGAGGCGCCAGTAAGAAACGCAAGAAAACTGACACCAAGTCCAGCGCCCAGGT
TACGAAGTGCCTTAATGAGAAGAGACGACGTGATCAAGAGAACACATTCTATGAAGAGCTGGCTGAGCTG
ATTGCTGCCAGCTTTGCTGACATGAACTCACTCTCTGTGAAACCGGACAAGTGCGCCATCCTCCAGGAAA
CTGTAACCCAGATACGCCGAATTAAGGAACAGGAGGGTGCGAGCAGTGATGCCATTCAGCAGGGTGAAGT
TTCATCATCAAAGCCTACTATTCTAGCTAATGAAGTACTTGGTCCACTACTGCTGCAGGCTCTTGATGGA
TTTTTATTTGTTGTGAACAAAGACTGCCATGTGGAGTACGTCTCGGAGAATGTGGAGCAGTTTATTAAGT
ACACCAAGGAAGATATTCTTGGGAAAAGTATTTATAATTTCATGCATGTGGGAGACACAACACGTTTCAG
TTCCATACTTTTGCCAATTACTTCCACAGCTTGGTCTGGCGAACATCAACCTCACAGGAACCGTACAATT
AACTGCCGCTTGTTAATCAAACCCCCAGATGATGCTGATGAAACTATGGAAGAGAAACAACAGCGTATAA
GCAAGTATGAGTATATGCAGATCTCATCTACTTTGCTTCCGTATCCAAGTGATCGTTCTGAGAGTGGTGA
CCTGAGTGAGTCAATTGATAGTAGCCCGTGTCTTATGTGTGTTGCTCGTCGTATACCACAAAATGAAAAA
TCTCTCCCCAACGCAGTTGAACAGTTCAGCATGAAGCTTGATGTGCATGGCAAAATAGTAGGAGTTGACA
CAACAAATATTTCACCTAGTTACTCGCAGTTTTTAAACAAGGATCTGGCAGGCCGTGTCATACAGGATCT
TTGCCATCAGCAAGACTTGCAGAAGCTGACAAGTCATTTAAGGGAGGCTGTGCAAACTGGTCACAATACA
AGTAAAATATACAGACTACGTGTGAAGACTCCTGATAAGTACATTCAAGTTCAGACAAAATCAAGGCTAT
TTCAGAACCCTAATGACCAGGAGGATTTCATCATGGCAACACACTCTATTATTGGAGACTCTGAGAACCC
TCTTCCGGAGTGTGGCCACCTCTCCAACTCTC
