# Supplementary material for: Crucial Role of Juvenile Hormone Receptor Components Methoprene-Tolerant and Taiman in Sexual Maturation of Adult Male Desert Locusts
Source: Biomolecules. 2021 Feb 9;11(2):244. doi: 10.3390/biom11020244 (PMC7915749; doi:10.3390/biom11020244)
Supplement: Supplementary file 1 [file biomolecules-11-00244-s001.zip › SupplementaryFiles/SuppFig2_Met.pdf]

```
              cov     pid    1 [         .         .         .         .         :         .         .         . 80
 1 SgMet    100.0%  100.0%     --------------------------------------------------------------------------------
 2 LmMet     98.1%   84.5%     --------------------------------------------------------------------------------
 3 BgMet     51.3%   27.5%     --------------------------------------------------------------------------------
 4 PkMet     84.3%   16.3%     ---------------------------------------------MGWTHTATAPPPSTGIAFSSGNSRSHSHSHGHNNG
 5 TcMet     45.3%   19.7%     --------------------------------------------------------------------------------
 6 HaMet     42.5%   14.0%     --------------------------------------------------------------------------------
 7 DmMet     58.5%   17.5%     --------------------------------------------------------------------------------
 8 DmGCE     63.1%   13.8%     MEGASRSRNSSTSHSQGRGQDIEDLKQDIPYFDEPPALDADLLVLGKSECQLDELAWDRDADGDADAPLETAPAVDLEED
 9 AaMet     72.5%   17.6%     --------------------------------------------------------------------------------
10 DmaMet    64.2%   15.9%     --------------------------------------------------------------------------------

              cov     pid   81         .         1         .         .         .         .         :         . 160
 1 SgMet    100.0%  100.0%     --------------------------------------------------------------------------------
 2 LmMet     98.1%   84.5%     -----------------------------------------------------------------------------MIS
 3 BgMet     51.3%   27.5%     ----------------------------------------------------------------------AETDEAEVLG
 4 PkMet     84.3%   16.3%     GSAARSTSSMYTSYGGASGYPSMATTAENVGLAAMGRTTDSASATAYRMTPSLQALPPPPSPSSPSTTSSSSPLMYPVQT
 5 TcMet     45.3%   19.7%     --------------------------------------------------------------------------------
 6 HaMet     42.5%   14.0%     -------------------------------------------MTSSGGASTHLGMHSAMASQIGLGAPPNEFGLSVGVP
 7 DmMet     58.5%   17.5%     ------------------------------------------------------------------------------MA
 8 DmGCE     63.1%   13.8%     NYPDENESSVLGSDYAPSGSGSGANSFYQSPTPSATGSGCDLMLRPPSNSMYHFNYRSPGSPMPVAPGVTNSRGLHPYAH
 9 AaMet     72.5%   17.6%     -------------------------------------------------------------------MQRSGPSSVATTT
10 DmaMet    64.2%   15.9%     --------------------------------------------------------------------------------

              cov     pid  161         .         .         .         2         .         .         .         . 240
 1 SgMet    100.0%  100.0%     --------------------------------------------------------------------------------
 2 LmMet     98.1%   84.5%     WEHYVP-------------------PPPPPD--------------------VYHELCPV---------------------
 3 BgMet     51.3%   27.5%     ADEVSE-------------------NTAPTG--------------------TTTSANQVQQQQQSQPQQQPL--------
 4 PkMet     84.3%   16.3%     SPHDYNHHLRNPYGYIDCRTAAVAAAAAAAN-------------------MPQSSSSSLYMAHNEYPPMTAN--------
 5 TcMet     45.3%   19.7%     -MDSGD-------------------CETEED--------------------SAPEACVSPEGSLLPSN------------
 6 HaMet     42.5%   14.0%     APTMGY----------------DIQATHSGD--------------------SSSDSHSPGMPTAKASD------------
 7 DmMet     58.5%   17.5%     APETGN--------------TGSTGSAGSTG--------------------SGSGSGSGSGSSSDPAN------------
 8 DmGCE     63.1%   13.8%     SPAHGNPPGFYPNMWYPNAPYGSAGAAGSAGGAVSGGRYMGYGPGGVPGGTNSGPGAGPGAMQAAYPGHSAHMHALHHQY
 9 AaMet     72.5%   17.6%     ATEPGE----------------KLDASTSAG--------------------NNSASKYDCEDSKAACKILN---------
10 DmaMet    64.2%   15.9%     ---------------------------------------------------------MDKMSETLPSS------------

                                                                       bHLH
              cov     pid  241         :         .         .         .         .         .         .         . 320
 1 SgMet    100.0%  100.0%     ----------------------------------------MRNKAEKQRRDKLNSLITELAHLVPMVAQSPRRMDKTSIL
 2 LmMet     98.1%   84.5%     -----------------------------------SNNRQMRNKAEKQRRDKLNSLITELAHLVPMVAQSPRRMDKTSIL
 3 BgMet     51.3%   27.5%     ----------------------------------PSYSRQNRNMMEKHRRDKMNAHISNLALLVPTVANSPKKMDKTSIL
 4 PkMet     84.3%   16.3%     ------------------------------------SNRMMRNLAEKMRRDKLNTYINELSTLVPMVSNSPKKMDKTSVL
 5 TcMet     45.3%   19.7%     -------------------------------------SREMRNRAEKMRRDKLNSYIGELATLVPMVARSAKRMDKTSIL
 6 HaMet     42.5%   14.0%     --------------------------------------RESRIIAEKQRRSQYNSQIGLIMSLLSDIVHSQRKVDKTSVL
 7 DmMet     58.5%   17.5%     -------------------------------------GREARNLAEKQRRDKLNASIQELATMVPHAAESSRRLDKTAVL
 8 DmGCE     63.1%   13.8%     PQPHPAHHPQHPHHSPHPHHPHPHETMMEMFQLSNSGREARNRAEKNRRDKLNGSIQELSTMVPHVAESPRRVDKTAVL
 9 AaMet     72.5%   17.6%     -------------------------------------GREARNRAEKNRRDKLNGSIQELSGMVPHVAESPRRVDKTAVL
10 DmaMet    64.2%   15.9%     -------------------------------------SREMRNRAEKQRRDKLNAYISELYSLVPSAAAAPRKLDKTSTL

                                  bHLH                                                       PAS
              cov     pid  321         .         .         :         .         .         .         .         . 400
 1 SgMet    100.0%  100.0%     RLSATFLRMYRCLPLKPGE-IHEPFLPFLETLKVS---------QVLLDFLQGMEGFMLVVTGA-GKIVFVTHTVEKLLG
 2 LmMet     98.1%   84.5%     RLSATFLRMYRCLPLKPGE-MHEPFLPFLETLKVS---------QVLLDFLQGMEGFMLVVTGA-GKIVFVTHTVEKLLG
 3 BgMet     51.3%   27.5%     RLTAAFLRLHKFLVADPGDPNRIEFPDFLKDCNLS---------QAFMELLQVMDGFLIIVSST-GNILFVSHTVETLLG
 4 PkMet     84.3%   16.3%     RLATTYLRIHRVTEATRDQRNVLPQILSSS--------------EWPQTLLDKMDGVMLIVTSA-GKIVFISHNIEKVLG
 5 TcMet     45.3%   19.7%     RLAATHLRIYQTLLSGKNHPHIQLPKHVDQY-------------LLEQLVCEQLGGFLLILTPN-GKIVFVSHTVEHLLG
 6 HaMet     42.5%   14.0%     RLAANKLRNEHVFGDTIKCGHIETWS------------------SSFLKFFDLFGGIMFGVTCR-GRLFIVSPNVQEKLG
 7 DmMet     58.5%   17.5%     RFATHGLRLQYVFGKSASRRRKKTGLKGTGMSASPVGDLPNPSLHLTDTLMQLLDCCFLTLTCS-GQIVLVSTSVEQLLG
 8 DmGCE     63.1%   13.8%     RFAAHALRLKHAFGNSLMQQRP----------------------QITDTLMDMLDSFFLTLTCH-GHILLISASIEQHLG
 9 AaMet     72.5%   17.6%     RFSAHALRLKYVFDTEQEQTKQEPSENAAG-----------QKTEVHDALFRMLNGFLLTVTCR-GQIVLVSASVEHFLG
10 DmaMet    64.2%   15.9%     RLSANFLRIHQNMDLRMKPYNRWNALAG-------------------HSILEKLDSFLLVVSCCSGKIIYVTDRVEKLLG

                                          PAS
              cov     pid  401                               .         :         .         .         . 480
 1 SgMet    100.0%  100.0%     HTQNDLLGQSLYNITRPEDHEELRRNIETAPSDGRASSAASGCSPALTSDESSCEDGAAGGS---------TPSSSPSAS
 2 LmMet     98.1%   84.5%     HTQNDLLGQSLYNITRPEDHEELRRNIETAPSDGQGR-AAAGCSPALTSDESSCEDGAAGGS---------TPGSSPSAS
 3 BgMet     51.3%   27.5%     HQQNFLLGQKLHSITCREDHDKLNKNLTPDPDPSAGDQESTG---QMSVGEDSSSSGDASSS---------SSQSLPSPQ
 4 PkMet     84.3%   16.3%     HTQTELMGHGLSNITFPPDLPELRRCLTPDKEDENCSDNTNANN------------------------------------
 5 TcMet     45.3%   19.7%     HLQTDLMGQSIFNITSPDDHDRLRMYINTESVLDG---------------------------------------------
 6 HaMet     42.5%   14.0%     YCHIDLLGQDMYHYVHPDDKETLRHHIYPQELQT----------------------------------------------
 7 DmMet     58.5%   17.5%     HCQSDLYGQNLLQITHPDDQDLLRQQLIPRDIETLFYQHQHHQQQGHNPQQHSTSTSASTSGSDLEEEEMETEEHRLGRQ
 8 DmGCE     63.1%   13.8%     HCQSDLYGQSIMQITHPEDQNMLKQQLIPTELENLFDAHGDSDAEGEP------------------------------RQ
 9 AaMet     72.5%   17.6%     HCQTDLYGQNLFNLIHPDDHNLLKQQLVPNNLVNLFDSAVSAPSTSRTSSG---------------------TETSAEEQ
10 DmaMet    64.2%   15.9%     HAQVDMMGYQLSCFVHQADQDAIEKRLSAFAAQVAANPDASDS-------------------------------------
```

```
              cov     pid    481         .         5         .         .         .         .         :         . 560
 1 SgMet   100.0%  100.0%    RPGTSASTASVGSSCDGGRAGLGGLFGSAGAAEHHRRSFYIHLAQRAGGRAAGSGSATAG--AGGGGGGGGG---AAGQG
 2 LmMet    98.1%   84.5%    RPGTSASTASVGSSSDGGRTGPGGPFGSAG-AEHLRRSFYVHLAQRAGGRAASSASGTGGSSGGGSGGGGGSVGVAGQG
 3 BgMet    51.3%   27.5%    HQQQSAPSSSTSTAVAVPEAAPSEPL-------RQRRSFYLRLSQKAVSRS-----------------------------
 4 PkMet    84.3%   16.3%    ----------------------------CTLVERQRRSFCLRLRHRCLARSEQP--------------------------
 5 TcMet    45.3%   19.7%    ---------------------------------DWKKCFNIRLKRAGPRTESA---------------------------
 6 HaMet    42.5%   14.0%    --------------------------SCDSRLFEHHHNFHIRMMRAGARSDPA---------------------------
 7 DmMet    58.5%   17.5%    QGEADDDEDHPYNRRTPSPRRMAHLATIDDRLRMDRRCFTVRLARASTRAEATR--------------------------
 8 DmGCE    63.1%   13.8%    RSKAEED-------------------AIDRKLREDRRSFRVRLARAGPRSEPT---------------------------
 9 AaMet    72.5%   17.6%    QRKSQDEED-----------------EIDRKLRQDRRKFTLRIARAGPRSEPT---------------------------
10 DmaMet   64.2%   15.9%    -------------------------------ADGQVYSFECRLAGRQLSRGEPT--------------------------

              cov     pid    561         .         .         .         6         .         .         .         . 640
 1 SgMet   100.0%  100.0%    AEQTTYELVHFLGYLQVPRSHEP-PPATHRPR-RHRD---TVPS------------------------------SNSDNV
 2 LmMet    98.1%   84.5%    AEQTTYELVHFLGYLQVLRSHDP-PPATHRPR-RHRD---TVPS------------------------------SNSDNV
 3 BgMet    51.3%   27.5%    -EITQYELMHVLGHLRVPQRPNPGPSAQSRTRNRQREGSSTGSS------------------------------ADSDTV
 4 PkMet    84.3%   16.3%    ----QYEPMHLNGFLRVPKKYTCQAQKKSRKCKGEMTATNSN-----------------------------------EIL
 5 TcMet    45.3%   19.7%    ----VYEPVRIMGVHRPGFDNDCNK------------NTSTS-----------------------------KEIALNNDV
 6 HaMet    42.5%   14.0%    ----RYERCRIDGILRRSDKATSNGVQDEQIIRRQRVRTNRT-----------------------------FSSSGNDVV
 7 DmMet    58.5%   17.5%    ----HYERVKIDGCFRRSDSSLTGGAAANYPIVSQLIRRSRNNNMLAAAAAVAAEAATVPPQHDAIAQAALHGISGNDIV
 8 DmGCE    63.1%   13.8%    ----AYEVVKIDGCFRRSDEAPRGVRSNHFSSNLQLIRRTRG-------------------RDDVIP---LHTISGNDII
 9 AaMet    72.5%   17.6%    ----AYELVTIDGFFRRADAAPRGERPSG-PSGLQLLRRARG-------------------RDDGIT---LQSINGNDIV
10 DmaMet   64.2%   15.9%    ----VYERVSVSGTFRGPRRRRDWADLKSSDRSVATVQQHNDYS---------------------------------EPL

                                                                          PAS-11
              cov     pid    641         :         .         .         7         .         .         .         . 720
 1 SgMet   100.0%  100.0%    LVAVVQLFREKKVMELS----------LLEATLDEYQTRHLKDGSIVYSDHRISVVAGYLADEVHGHNAFTFMHQDDMAY
 2 LmMet    98.1%   84.5%    LVAVVQLFREKRVMELS----------LLEATLDEYQTRHLKDGSIVSSDHRISVVAGYLTDEVHMQNAFTFMHHDDMAY
 3 BgMet    51.3%   27.5%    LIAVVRTVRESSLVERS----------LLEPSKDEYVTRHLIDGRIIYSDHRISVVAGYMAEEVAGLSAFKFMHKDDMRF
 4 PkMet    84.3%   16.3%    LVAVMKPAR---EMTLMS---------IVEASQEEYVTRHWYDGRIVFCDQRISLVSGYLPEEVKGLQALQFMAKEEVHW
 5 TcMet    45.3%   19.7%    LLFFVKVFR---PEPLCER--------LFEASREEYVTRHLIDGRIIGCDQRISFIAGYMTEEVSGLSAFKFMHREDVRW
 6 HaMet    42.5%   14.0%    FIGMIHVLSSAMPPRILP-----------PTAYSEYWTRHLIDGRIVQCDQSISLAIGYMTEEVTGTSAFVFMHKDDVRW
 7 DmMet    58.5%   17.5%    LVAMARVLREERPPEETEGTVGLTIYRQPEPYQLEYHTRHLIDGSIIDCDQRIGLVAGYMKDEVRNLSPFCFMHLDDVRW
 8 DmGCE    63.1%   13.8%    LTGCARIIR---PPKIAS--------RLIDANTLEYKTRHLIDGRIIDCDQRIGIVAGYMTDEVRNLSPFTFMHNDDVRW
 9 AaMet    72.5%   17.6%    LVAVARVQK---VPTICD--------RLIEACRYEYKTRHLIDGRIVQCDHRISVVAGYLTTEVSGLSPFTFMHKDDVRW
10 DmaMet   64.2%   15.9%    FIGLVRILQTPNTLPPLT---------IMQAVQDEYITQHTTGGTIIQTDHRIAIIAGYLSGEVTGMSAYDYVYSEDLEY

              cov     pid    721         .         .         :         .         .         .         .       8 800
 1 SgMet   100.0%  100.0%    ALIALQQMFDK-NEPFGSSTYRLVTKNGQYIYMRTRGYLEFAHGSKDVETFLCINTLLSKEEGEDGLREMKQRFSAYVSA
 2 LmMet    98.1%   84.5%    ALIALQQMFDK-NEPFGSSTYRLATKNGQYIYMRTRGYLEFAPGSKEVETFLCINTLLSKEEGEEGLREMKQRFSAYVSA
 3 BgMet    51.3%   27.5%    TMIALRQMYDRGKDSYGSSCYRLQSKTGQYIYLRTHGYLEYDKDTQQIVSFICINTLLTEEEGERRIQEMKARFSATIT-
 4 PkMet    84.3%   16.3%    VMIALRQMLWN-REGFGKSCYRLKRKNGDFIYMVTHGYLEP-CEDLQKSTFVCVNTLVSSEEGLREIRNMKKKFTPIIER
 5 TcMet    45.3%   19.7%    VMIALRQMYDR-GESKGSSCYRLLSRNGQFIYLRTFGFLEIDDQG-TVESFVCVNTLVSEQEGLQLINEMKKRYSALINS
 6 HaMet    42.5%   14.0%    VICVLRQMYDE-SREFGESYYRLMTRSGHFIYMRTRGFLEIDRATKKVQSFVCVNSVIGEDYGCRMMDEMKRKYSVIIDV
 7 DmMet    58.5%   17.5%    VIVALRQMYDC-NSDYGESCYRLLSRNGRFIYLHTKGFLEVDRGSNKVHSFLCVNTLLDEEAGRQKVQEMKEKFSTIIKA
 8 DmGCE    63.1%   13.8%    VIVALRQMYDC-NSSYGESTYRLFTRNGNIIYLQSKGYLEIDKETNKVHSFVCVNTLLGEEEGKRRVQEMKKKFSVIINT
 9 AaMet    72.5%   17.6%    VIVALRQMYDY-SQNYGESCYRLMTRTGDFVYLKTRGYLEVDDSSKKVQSFVCINTLVSDEEGRRLVREMKHKFSVIVEA
10 DmaMet   64.2%   15.9%    TLKAQSLMLTR---SEGMVTYRLKTSTGRLIFLRSRGFIQYDENTKEIVSFFCINSLIDEEQGMKEMQEMRAMLDKLNIA

              cov     pid    801         .         .         .         .         :         .         .         . 880
 1 SgMet   100.0%  100.0%    LRAAGADLSLAGIAAAMSAAPPPPR--MVKGSELPKLPPPPPPPPPSLQPSSPSPLPPPRPPPPPPPQPPAAITSLSSPE
 2 LmMet    98.1%   84.5%    LRAAGADLSLTGIAAAIAAAPPPFRSSAVTASERPTLAPPPPPPPPPLQP-------PPPPPPPPPPAPPTAANSLSSSE
 3 BgMet    51.3%   27.5%    --------------------------------------------------------------------------------
 4 PkMet    84.3%   16.3%    RDEPAIAAIAEKYAFVRMQ-------------------------------------------------------------
 5 TcMet    45.3%   19.7%    QSCPITS-------------------------------------------------------------------------
 6 HaMet    42.5%   14.0%    DKQQTE--------------------------------------------------------------------------
 7 DmMet    58.5%   17.5%    EMPTQ---------------------------------------------------------------------------
 8 DmGCE    63.1%   13.8%    QIP-----------------------------------------------------------------------------
 9 AaMet    72.5%   17.6%    DELPD---------------------------------------------------------------------------
10 DmaMet   64.2%   15.9%    TVTPAIAS------------------------------------------------------------------------

              cov     pid    881         .         9         .         .         .         .         :         . 960
 1 SgMet   100.0%  100.0%    IITNSTLCEVSPPFTAMVTESAASSEQSADVFGSVSRSEAAASKYRNQAPTAASRSPTVDPY------------------
 2 LmMet    98.1%   84.5%    IISNTTVCEVSPPFTAMVTESTPSSEQGADVFGSVSPSEAAASKYRNHTPTTASRSPTADPY------------------
 3 BgMet    51.3%   27.5%    -----------------------SSRQQAAILG----------------PSTSTSVGNVD--------------------
 4 PkMet    84.3%   16.3%    ----------GRNVPDLNPTTEDPDELKNALKAMVTNIPPPIEVKEDPEQCRETQFAKVKYISKTLPPPTIPTG------
 5 TcMet    45.3%   19.7%    ----------SGSTDSSSQSVEDPQQVEAAIVHLIANLPSPG--------------------------------------
 6 HaMet    42.5%   14.0%    ------------PPPIDDAPVEHPKRLERIVMHLVEPSVNEN--------------------------------------
 7 DmMet    58.5%   17.5%    ------------SSSPDLPASQAPQQLERIVLYLIENLQKS---------------------------------------
 8 DmGCE    63.1%   13.8%    ------------QSTIDVPASEHPALLEKAVLRLIQNLQKSGENGGHDDGDEDDDAQDGDDD------------------
 9 AaMet    72.5%   17.6%    --------------ESDEPAVENPTQIEKAVLNLLTNLHSEDDEPSERALPSNTSQETDGSEGSQLAIIAPSSKAVKSAI
10 DmaMet   64.2%   15.9%    ------------SPTSTIEPVGAASTQEPLSRCVRASVGKPPPSFSSNGCHLANGSRVSG--------------------
```

```
             cov    pid  961       .         .         .         0         .         .         .         .  1040
 1 SgMet   100.0% 100.0%     ----------------------------------------------------------LPAARPRAVSEGDDTTGFNLQN
 2 LmMet    98.1%  84.5%     ----------------------------------------------------------LPAARPRAVSEGEDTTAISLQN
 3 BgMet    51.3%  27.5%     --------------------------------------------------------------------------------
 4 PkMet    84.3%  16.3%     ------------------------------------------------------------KVITVMDEKTASPTSPAPST
 5 TcMet    45.3%  19.7%     --------------------------------------------------------------------------------
 6 HaMet    42.5%  14.0%     --------------------------------------------------------------------------------
 7 DmMet    58.5%  17.5%     -------------------------------------------------------------VDSAETVGGQGMESLMDDG
 8 DmGCE    63.1%  13.8%     -----------------------------------------------------------EEDDDDDQDDGARSMSEFGDP
 9 AaMet    72.5%  17.6%     VKSMNVIAIASKKMSNGSNSPLRDSTGASPRSKDHSGSPSAIITMQRPSVLQKAHSPALESRVPPLHGTDHDFIQPFSPA
10 DmaMet   64.2%  15.9%     --------------------------------------------------------------LPRSSIMPNGHNSLCISP
```

```
             cov    pid 1041       :         .         .         .         .         1         .         .  1120
 1 SgMet   100.0% 100.0%     FVSLFKVPEEEENSVEVQLSPLANSTGITTDLEEKEQLRNPLSPSSPPSRHPYTAAQRIRAASESELQTANSGSTFIQSD
 2 LmMet    98.1%  84.5%     FVNLFKVPEEEEHSVDVQRSPLVNNTGVISDLEEKPRITNSLSPSSPPSRHPYAAAQRIRAASESELQAANSGSTFIQSD
 3 BgMet    51.3%  27.5%     -----------NDQLEVAISQLISNIPEVRDDNKE--------PSNLPDTQYAKAVRYSKVLPSATEQAAKVGIKPVIPN
 4 PkMet    84.3%  16.3%     SSKRDRLSPLPVSTSAAYGTTATNSASMGSPSPSPSPSPSSSSQCQHAVVSPSSMAALSPCGAALSRTSFQNRPSVLRI
 5 TcMet    45.3%  19.7%     -----------------SDQRSTPSPRVYGNVNENQDCSTPTENSPTKPYYKLKTANNKRPPSTELGTNIYTSSKRQRTS
 6 HaMet    42.5%  14.0%     ------------------------IDELQLVPPSKENIISAIQNS--EKVVQETGVRFDSRKRRKVDCDDN---------
 7 DmMet    58.5%  17.5%     YSSP-------ANTLTLEELAPSPTPALALVPPAPSSVKSSISKS--VSVVNVTAARKFQQEHQKQRERDREQLKERTNS
 8 DmGCE    63.1%  13.8%     YGSHHGRSHHGSSALSSHGHGNAKTPPLALVPPEASSVKSAITKS--ISVVNVTAAKHLRGIHASTAVKSPSSLGSCTCS
 9 AaMet    72.5%  17.6%     GSTSSSSSSSGSVFSPASHQRNSRSPFGGSIPPTSPPAIAATPSTSVSRIVDNTASGHSRSISVLKRTHSNSCLDSSDGS
10 DmaMet   64.2%  15.9%     SSELQYSPSGSSSSSFSEERCQSSTPHSLVSSPMEIPNLVAVSYPFAPIPFPWRPSTDCSPITTTSNGHVNTREVSKSPD
```

```
             cov    pid 1121       .         .         :         .         .         .         .        2 1200
 1 SgMet   100.0% 100.0%     PASRGGNWQFPRPGDMPHSGTTERRPPERPTVITGRCCAGSERMKCSRLSVMSVTGDEKRITSVRGEVTVLKRTMSADVE
 2 LmMet    98.1%  84.5%     PADRGCSWQFQRHGDVSHSGTTDRRPPERPTVITGRCCAGSERMRCSRLSIMSVTGEEKRITSVHGEV-VLKRTMSADEE
 3 BgMet    51.3%  27.5%     ----------ARTGPISPTGPLTVTIPEPPQRIHP----------------------EATWTSIKRES------------
 4 PkMet    84.3%  16.3%     APARQAVNSVSATVADNYAISSTSNFDGDKATNDDAGRKYSASFCGSPAAAAVAGTGSVRRGSTNCDFIKQECAIKQEAV
 5 TcMet    45.3%  19.7%     P-------------------------------------------------------------------------------
 6 HaMet    42.5%  14.0%     --------------------------------------------------------------------------------
 7 DmMet    58.5%  17.5%     T--------QGV----IRQLSSCLSEAETA----------------------------------SCIL------------
 8 DmGCE    63.1%  13.8%     DSHSPCDFCQGAPTTDLQAVGSNLKRGSTAHVETEEKLSKRRFIPSTEIEHVLHTSLDQIGRNLTQQL------------
 9 AaMet    72.5%  17.6%     SIGHTADFVVQKRPKINTILDLDTCRTRLSNPATDLITMLPQAFHAVDQSLLKVDDDTAKLRAQVSGY------------
10 DmaMet   64.2%  15.9%     VDQSDPLGEPGLLALTLDHQWETAPNSQSMIVQHNG--------------------------------------------
```

```
             cov    pid 1201       .         .         .         .         :         .         .  1280
 1 SgMet   100.0% 100.0%     NPSTTKKLNVGPRYCRQRHRVEHPPTEALSVEVNTLPPDELQHIILSRDSGMYNSQPTVLVSQHSLFR----------TS
 2 LmMet    98.1%  84.5%     NPSCTKKLNVGPRHCRQRHRIEHPPTEALSVEVNTLPPDELQHIILSRDS----SQPTVLVSQHRPFR----------TS
 3 BgMet    51.3%  27.5%     -------------------------------VITSLVP------------------------------------------
 4 PkMet    84.3%  16.3%     DVDNYNDDGCGVDDDDDDADADADDDDLEAQIAAVIQTDSGQQLVAAMAAAQAMPRVGVVVSGRSALKRRLDCDASSFAS
 5 TcMet    45.3%  19.7%     -------QLSPMSSLPPYPNRTQITEVTQQTEPSIYQHDQLLRNKV----------------------------------
 6 HaMet    42.5%  14.0%     -----------------------SEYLKRHSGLQDVNVDL----------------------------------------
 7 DmMet    58.5%  17.5%     -SPASSLS-ASEAPDTPDPHSNTSPPPSLHTRPSVLHRTLTSTLR-----------------------------------
 8 DmGCE    63.1%  13.8%     -NVARNLREQSQRYELPHANQRFDEIMQEHQKQSELYVNIKSEYEVQLQHK----ASTRKSSDSDRNQ----------EQ
 9 AaMet    72.5%  17.6%     -ENLQGKSLQQLDSIVEASHEQRNVLQSIKQELTQFQSNNGSNDQDGAGGRGPIVVNDLLPGGGAIVD----------DL
10 DmaMet   64.2%  15.9%     -PQSHQQSLVAVSPLQAHLLQSSPNDNIEPIRSTLKSPPSSTSCPVQPPHQYQEDALKISIPDRCPTN------------
```

```
             cov    pid 1281       .         3         .         .         .         .         :         .  1360
 1 SgMet   100.0% 100.0%     QPALQTVTQNVPASSLSPPQLAEMQSTSVTPPSFIMEEPSGLPLVNLSDANTLSVGCQDPLISPDHGDSDSFSSLGLPDP
 2 LmMet    98.1%  84.5%     QPALQTVTQNVPAVSLSPPQLADIT-----PPPFVMEEPGGMPLVNLSDT-TLSVGCQDPLISPDHGDSDSFSSLGLPDP
 3 BgMet    51.3%  27.5%     --------------------------------------------------------------------------------
 4 PkMet    84.3%  16.3%     SIVPIKKNAHCADGVAKARHQKHLLAATAVPSPLPLPPSHQVSSSSSSYVPYSAAAAVAASASPCSNSAGSALLLATTQI
 5 TcMet    45.3%  19.7%     --------------------------------------------------------------------------------
 6 HaMet    42.5%  14.0%     --------------------------------------------------------------------------------
 7 DmMet    58.5%  17.5%     --------------------------------------------------------------------------------
 8 DmGCE    63.1%  13.8%     PPPPLQEDDQD---------------------------------------------------------------------
 9 AaMet    72.5%  17.6%     DPPSLMMEDFPSDSSNLMADLDNNGSLLEGDSPLEFFSSINTPLISPTTATASVITSSRSVGGTGTGGASGTAGSFYQGA
10 DmaMet   64.2%  15.9%     ---ELSRNNCLNGYIIWPQKMSPKNRKENLILSYLDVDKHAYKQPEITGTADQHYYHSDLKVNAGTNSA-----------
```

```
             cov    pid 1361       .         .         .         4         .         .         .         .  1440
 1 SgMet   100.0% 100.0%     ENNPEVLDLLEASELKLNFDTLQEVRSSNEPEAPSGPETAVTLER--FLARKHLQLESCMQRQETQMHCIEHDLTQLPLA
 2 LmMet    98.1%  84.5%     ENNPEVLDLLEAPELKLNFDTLQEARSSNDPEAPTGPETAATLER--ILARKHLQLESCMQLQETQMHCIEHDLTQLPLA
 3 BgMet    51.3%  27.5%     --------------------------------------------------------------------------------
 4 PkMet    84.3%  16.3%     SRIDDPYTRLADIDVCDPTAQFVHNITADSFETTNQPEQVLQLLDNENLKHRHIELESKMHIQGAQIHAIEGDLDKVPFT
 5 TcMet    45.3%  19.7%     --------------------------------------------------------------------------------
 6 HaMet    42.5%  14.0%     --------------------------------------------------------------------------------
 7 DmMet    58.5%  17.5%     --------------------------------------------------------------------------------
 8 DmGCE    63.1%  13.8%     --------------------------------------------------------------------------------
 9 AaMet    72.5%  17.6%     DLNPS---------------------------------------------------------------------------
10 DmaMet   64.2%  15.9%     --------------------------------------------------------------------------------
```

```
             cov     pid  1441          :         .         .         .         .         ] 1499
 1 SgMet   100.0% 100.0%       DHVFRSNLTQLQASGPIHARKEERQASKLHIPVLTPIFMRNNIRSTRYC----------
 2 LmMet    98.1%  84.5%       DHVFRSNLTQLQASGPIHARKEERQASKLHIPVLTPIFMRNNIRSTRYC----------
 3 BgMet    51.3%  27.5%       -----------------------------------------------------------
 4 PkMet    84.3%  16.3%       TEQLGFQTDLTQLKEQHVKQKKMLMTLQQDHHSLQHCNTTDGCANITSNTTPTHQNVGV
 5 TcMet    45.3%  19.7%       -----------------------------------------------------------
 6 HaMet    42.5%  14.0%       -----------------------------------------------------------
 7 DmMet    58.5%  17.5%       -----------------------------------------------------------
 8 DmGCE    63.1%  13.8%       -----------------------------------------------------------
 9 AaMet    72.5%  17.6%       -----------------------------------------------------------
10 DmaMet   64.2%  15.9%       -----------------------------------------------------------
```
