# Supplementary material for: Crucial Role of Juvenile Hormone Receptor Components Methoprene-Tolerant and Taiman in Sexual Maturation of Adult Male Desert Locusts
Source: Biomolecules. 2021 Feb 9;11(2):244. doi: 10.3390/biom11020244 (PMC7915749; doi:10.3390/biom11020244)
Supplement: Supplementary file 1 [file biomolecules-11-00244-s001.zip › SupplementaryFiles/SuppFig4.pdf]

MSTVIAENAGLGPCDLQDPVWVKMSAAVTGGASKKRKKTDTKSSAQVTKCLNEKRRRDQENTFYEELAELIAASFADM
NSLSVKPDKCAILQETVTQIRRIKEQEGASSDAIQQGEVSSSKPTILANEVLGPLLLQALDGFLFVVNKDCHVEYVSENVEQ
FIKYTKEDILGKSIYNFMHVGDTTRFSSILLPITSTAWSGEHQPHRNRTINCRLLIKPPDDADETMEEKQQRISKYEYMQIS
STLLPYPSDRSESGDLSESIDSSPCLMCVARRIPQNEKSLPNAVEQFSMKLDVHGKIVGVDTTNISPSYSQFLNKDLAGRVI
QDLCHQQDLQKLTSHLREAVQTGHNTSKIYRLRVKTPDKYIQVQTKSRLFQNPNDQEDFIMATHSIIGDSENPLPECGHL
SNSPRSATSAGQGNPSFGTSPSDGNGSVGGPLMSSAVNGQVSAVGSVGGRTNSSGFGSVTSDANSPAYHTYSTEIGLN
DFDFFPSSTWDIVGGGETSPDGSSVNAAQSGWDRRPDSRQSQSLTPVSTPNPRPPSGAGYSPSGGVCASPLNPYVHQT
SPAARVSTPAHPFANSFPFSPLQEQGPDGSFCSDGTEESKSGVQESANVNGSSDGGSAPGDSGRRLRNLLLTKRQSTSA
DEVSTGDAGGQVGDVDVRNRHRILKGLLNQDDDEDARSDEVPAGAGAAGRGSVGGRLQADAPRMSGHSSTGNNM
LLKLLNEKSDDDDLEKRAGLKKQNELLQQLLKEQDEESKPQVGPDTGQHRDDDPLLESLGFRNPSPSPPSGAGVGVDVT
GSLTVSSLIGGRKRPSDDGDESGVSSAKMRLGGPSGAPPGVSRSGTAAGTSKLWERNKMLASLLAKEPSQPATIPPIPAS
VISATPQEILPRVSKPQTPSWPSGNVQPPSSTAATARPTQQVTDVSRQQVRPQPNRPSATPFLNQMLTQQDHQQSN
MLLQQSIQQQKVQQQPSQQQQQQQAQQQQVQSSQQQAQQQVQQQQQQQQQQQQVQQQRGQLNQLE
SSFNIPVSGATTVDFRSVSGSGVSSPSTWDNQSSDPVLSDLLDQVIDIVPDDSSAIMNMLDAMESSPNNSGYQQGLNE
KMAIHAIQKSLMLCESAVKSPSSPTVSLPGTPPTYAASTMSNQTPQQGSFPPPPVYQRPRFGLPAGTTPVPGRPGAPQY
GIGVLNQQALIQQRKILQQQQQKQRLLQQQQQQQLLIPSNATADQLPTGLQNIDSLLNNTVAPNVSLQRSASVPDSQL
SPNFGGQVLNSAASQISPGQRLNQQPYSPHSQLASPLGQQQVFSPVSTGMNSFSSGQQAAQSRLSPHPPTGLPSFQQ
SQLSPRISQGQPGYPVLTQGRPFGPQRQQQQQQPSQQQQPQQLPPVRSLASPGGAVVGPRQSPYQAEQFPPPSSPG
TTTGFQAQGQFPQQLRLQRTISAPSATTQLPGGVNHSRLYGTKQELPHPALSPSEPVSPHPFHPHGPPLPDPHHHHPHP
LSHPHPHHHHHHIPHHQQQPQPQHHNPHQPSQHHPVMYQAQPRPPPDSQFCYDQPNFQLYGASERGRPQAHTPT
NHQPGGGGGGGGNGGMTSEYVRQELRAIVGARQSNGGSSGGVGMTVSGQQRQLQLGPGQSVSAADLEAFGFVCE
PPSTGTSDSPKLWGSIGSELSSGSPQAGFSSSRNPTEESPRPSDHKSSLLQKLLSE*
