# Supplementary material for: Crucial Role of Juvenile Hormone Receptor Components Methoprene-Tolerant and Taiman in Sexual Maturation of Adult Male Desert Locusts
Source: Biomolecules. 2021 Feb 9;11(2):244. doi: 10.3390/biom11020244 (PMC7915749; doi:10.3390/biom11020244)
Supplement: Supplementary file 1 [file biomolecules-11-00244-s001.zip › SupplementaryFiles/SuppFig5_Taiman.pdf]

```
           cov    pid     1 [       .         .         .         .         :         .         .         . 80
1 SgTai 100.0% 100.0%       --------------------------------------------------------------------------------
2 LmTai  99.9%  87.1%       --------------------------------------------------------------------------------
3 PkTai  84.4%  29.3%       --------------------------------------------------------------------------------
4 BgTai  90.8%  52.9%       --------------------------------------------------------------------------------
5 DmTai  92.7%  15.8%       MSIAAAENAGLPSPSDLPDHWASGTSNPSTSTSSSSSSISASGSNNNKYNSGNISVCNLPRSSPAAATAAVTLSSAAGGI
6 AaTai  87.1%  17.5%       ---------------------------MHFGQSSRSSTVHRCPYPNPTYLYG------------------ITFNSTSTNL
7 MnTai  90.8%  19.5%       --------------------------------------------------------------------------------

           cov    pid    81         .         1         .         .         .         .         :         . 160
1 SgTai 100.0% 100.0%       --------------------------------------------------------------------------------
2 LmTai  99.9%  87.1%       --------------------------------------------------------------------------------
3 PkTai  84.4%  29.3%       --------------------------------------------------------------------------------
4 BgTai  90.8%  52.9%       --------------------------------------------------------------------------------
5 DmTai  92.7%  15.8%       GSNLLSGLHHGLAPGTHPLQRATAASGGGAGAGGVAGASTAAALTLQQHQHQQQQQQQQQQAQHQHQHQQQTQQQQLA
6 AaTai  87.1%  17.5%       KQQRLG----------------------------------PCELPLPDHWLVHTQLSQSSSQQYSTPSPAIGGPSLGLGG
7 MnTai  90.8%  19.5%       --------------------------------------------------------------------------------

           cov    pid   161         .         .         .         2         .         .         .         . 240
1 SgTai 100.0% 100.0%       ----------------------------MSTVIAENAGLGPCDLQDPVWVKMSAAVTGGASKKRKKTDTK---SSAQVTK
2 LmTai  99.9%  87.1%       ----------------------------MSTVIAENAGLGPCDLQDPLWVKMSAAVTGGASKKRKKTDTK---SSAQVTK
3 PkTai  84.4%  29.3%       ---------------------------------------------------MSVASSG--VKKRKKTENK---PQSQINK
4 BgTai  90.8%  52.9%       ---------------------------------------------------MSAAVSGGVNKKRKKTDTKPH-AQACINK
5 DmTai  92.7%  15.8%       YQHQQLQQQIISHRSIQNKAAATAAAQTANLAAALQRSAAIMNAVASPISANSANSATSGRKIRRKTDSKVNLPQSQINK
6 AaTai  87.1%  17.5%       LPPALPQQQSSSHS-------TSPLQQTPSASQQQQLQPKIMNAVVPAVSVPAAN-----KKIRRKPDTKP---QSQINK
7 MnTai  90.8%  19.5%       ----------------------------------------------------MSSPTQAVSKKRKKPDAK---PQSQINK

           cov    pid   241                       bHLH                                  3         .         . 320
1 SgTai 100.0% 100.0%       CLNEKRRRDQENTFYEELAELIAASFAD-MNSLSVKPDKCAILQETVTQIRRIKEQEGA---------------------
2 LmTai  99.9%  87.1%       CLNEKRRRDQENTFYEELAELIAASFAD-MNSLSVKPDKCAILQETVTQIRRIKDQEGA---------------------
3 PkTai  84.4%  29.3%       CLNEKRRREQENIFIEELAELISVSFSD-MNSLSVKPDKCAILQETVHQIRNIKQRQQQEQELQQQQN------------
4 BgTai  90.8%  52.9%       CRNEKRRREQENIFIEELAELISAGFPD-MSSLSVKPDKCAILKETVHQIQTIKQQEGS---------------------
5 DmTai  92.7%  15.8%       CNNEKRRREAENGYIEQLSEILTLNKRG--DMTSTKPDKAAILNQVVRTYREICDKGQNRDISSTSTNNNNSTTTTNNNT
6 AaTai  87.1%  17.5%       CNNEKRRRELENEYIEQLGEFLQINKR---DMTACKPDKAAILSEVVKTFRRLLEQG-NRDLTGSNR-------------
7 MnTai  90.8%  19.5%       CLNEKRRREQENTYIEELAELIQASIASDMSSLSVKPDKCAILQQTVNQIRRIKAAEGAG--------------------

           cov    pid   321         .         .         :         .         .         .        PAS        400
1 SgTai 100.0% 100.0%       --------------------------SSDAIQQGEVSSSKPTILANEVLG-------------PLLLQALDGFLFVVNKD
2 LmTai  99.9%  87.1%       --------------------------SSDAIQQGEVSSSKPTILANEVLG-------------PLLLQALDGFLFIVNKD
3 PkTai  84.4%  29.3%       ------------------QPNQQGVVSTDDIQQGEVSSSKPTVLSNAIFG-------------PLLLEALEGFAFLLNQE
4 BgTai  90.8%  52.9%       --------------------------SSDAVQQGEVSSSKPTILASEVFG-------------PLLLEALEGFLFVVNTE
5 DmTai  92.7%  15.8%       NSNNNNNTSKPQATSTRCSRCATDNCSIHPVQQGEVSSTEPPLPEPSLLLGQVPEISAYFEALEHYISGVGWVLLQVNAN
6 AaTai  87.1%  17.5%       --------------CSKCSPDCSDSCKLHPVQQGEVSSTEPPLPEP-SVNGHSPEKSAYFEAVKYYISNVGWVLLEINSE
7 MnTai  90.8%  19.5%       ---------------------------EGAVQQGEVSSSKPTLLPTQRIG-------------TLVLEALGGFLFIVSHD

           cov    pid   401                   PAS                           :         .         .         . 480
1 SgTai 100.0% 100.0%       CHVEYVSENVEQFIKYTKEDILGKSIYNFMHVGDTTRFSSILLPITSTAWS-----------------------------
2 LmTai  99.9%  87.1%       CHVEYVSENVEQFIKYTKEDILGKSIYNFMHVGDTTRFSSILLPITSTAWS-----------------------------
3 PkTai  84.4%  29.3%       GKVEYVTDNVSQFIKYSKDEILGKNIYSIVHPHDHGKFSSILLPTSVGNWSQSGS-------------------------
4 BgTai  90.8%  52.9%       GKVEFVTENVCHFIKYSKEDVLGQSVYNIIHLGDHARFSASLLP-TSIGWT-----------------------------
5 DmTai  92.7%  15.8%       GIIESCTQNIRDLIGYEKQELYHQPLYMYLYSGDHAKLEPIINTMYNNPNGGNSNSANNSGPGGSSAGTSAGVWGDLEEL
6 AaTai  87.1%  17.5%       GVIECATDNVLDVLHYTRTELHGQSIVSYLHTGDHSKLSPILNKNSFELNWDQNE-----------------VRISVVLL
7 MnTai  90.8%  19.5%       GKIEFVSDKVSHFIKFTSEDLTGTSIYNIIHVGDHAKFSSSFMSVGWPSEAS----------------------------

           cov    pid   481         .         5         .         .         .         .         :         . 560
1 SgTai 100.0% 100.0%       ----------------------GEHQPHRNRTINCRLLIKPPDDADETMEEKQQR-------ISKYEYMQISSTLLP---
2 LmTai  99.9%  87.1%       ----------------------GEHQPHRNRTINCRLLIKPPDDADETMEEKQQR-------ISKYEYMQISSTLLP---
3 PkTai  84.4%  29.3%       --------------------GSGANQQPKHRPFNCKFLIKYSDDSDESVVDNKEH-------TSNYDQVQISSTLSKGEG
4 BgTai  90.8%  52.9%       ----------------------PDPSPNRSRSFNCRFLIKPPDNQDETMEEKQQR-------VSKYETMQISSTLLP---
5 DmTai  92.7%  15.8%       NNGNASQGSNSSGAGGLGGAGGAAAGKKRSISTKVRMLVKDTRTATQTSSNCEEKPLRQSGHQDKYEEVVLIAAPVK---
6 AaTai  87.1%  17.5%       DLG-----------LTLPLHLQFLQPPKRTIRTKIRWLLKTPESANDTIEQKQQR-------QEKYKDLLIISAPVK---
7 MnTai  90.8%  19.5%       -------------------------EASRSRNFNCRLLVKPPDDQEETMEEKQQR-------VSQYENMQITAVLLP---

           cov    pid   561         .         .         .         6         .          PAS-11            640
1 SgTai 100.0% 100.0%       ------------------YPSDRSESGDLS-ESIDSSPCLMCVARRIPQNEKSLPNAVEQFSMKLDVHGKIVGVDTTNIS
2 LmTai  99.9%  87.1%       ------------------YPSDRSESGDLS-ESIDSSPCLMCVARRIPQNEKSLTNPVEQFSMKLDVHGKIVGVDTTNIS
3 PkTai  84.4%  29.3%       TYVSFQISSTLLKGDGVSGSNGGNGNNEISSECGEMGPGLICVARRIPTTEKHMSPAVEQFTTKLDPQGKIIGIDTSGVS
4 BgTai  90.8%  52.9%       ------------------YPNERTEGGDVTSESAEVGPCLMCVARRIPQNEKLTGPPFEQFTTKLDVTGKIIGIDTSGVS
5 DmTai  92.7%  15.8%       ---------------------DDADASSS-VLCLITRPEDESPLEINIQQHVQ-QQPIEQMTFKLDIHGKILTLDPTALR
6 AaTai  87.1%  17.5%       ---------------------DDTDAESSSVLCLITLPEDEHHQGPAIESHTMPQTLDEQLTLKLDMTGAVIDFDAATLR
7 MnTai  90.8%  19.5%       --------------------SSIVENKTCESFEGEGQQCLVCVVRRIPSNERIQGTPVEQFTTKLDTQGTIIGLDSSGVS
```

```
                                                          PAS-11
            cov     pid   641                                                                                720
1 SgTai 100.0% 100.0%   PSYSQFLNKDLAGRVIQDLCHQQDLQKLTSHLREAVQTGHNT-----------SKIYRLRVKTPDKYIQVQTKSRLFQNP
2 LmTai  99.9%  87.1%   PSYSQFLNKDLTGRVIQDLCHQQDLQKLTSHLREAVQTGHNT-----------SKIYRLRVKTPDKYIQVQTKSRLFKNP
3 PkTai  84.4%  29.3%   STYSLYLNKDLKGKALQELCYGTDLYNLTAHLKEVHQNEKAT-----------SKIYRLMIS-PEKYLHIQTNSRLFRAT
4 BgTai  90.8%  52.9%   STYSQYLNKDLMGCIIQELCHQRDLQKLENHLKEVLHTGQST-----------SAPYRLRVTTPDKYVHVQTKSKLFKSN
5 DmTai  92.7%  15.8%   EPFKQHLQTWVG-RLWQDLCHPHDLSTLKSHLRDIQDSASANSPGAGAGTSVVSRPFRLRLGAPDVYVHVKANSRLFLNQ
6 AaTai  87.1%  17.5%   KQFTDYLTKETV-RSIHDLCHFQDRPRLNEHLQNVMNANGAAQVSS----------YRLRLGGPDVYVHVKAQTRLFRNN
7 MnTai  90.8%  19.5%   PRYSQFLNKERIGEPYTKLIPPADISRVQGHLRETVQSGQAS-----------SQVYCVKIG-TERSIRVQTKSKFFRDL

                            PAS-11
            cov     pid   721                  :          .         .         .         .        8 800
1 SgTai 100.0% 100.0%   N-DQED--FIMATHSIIGDSEN-------------PLPECGHLSNS----PRSATSAGQGNPSFGTSPSDGNG-------
2 LmTai  99.9%  87.1%   N-DQED--FIMATHSIIGDSEN-------------PLPECGHLSNS----PRSATSAGQGNPSFGTSPSDGNG-------
3 PkTai  84.4%  29.3%   P-GSGEPDFIMAAHSIISEND------------------VCQLIESASLNPRQSSSLGGG-----PMMNALNG-------
4 BgTai  90.8%  52.9%   G-TLQDPDFIMATHSIINDTEI-------------APAECGHLSNSQTNSPRGGSMLSTGSVASGPSSNGNSN-------
5 DmTai  92.7%  15.8%   --TPGEGDFIMSVQTLLNSENDMNSSNTGAGSGGLGLGQLCAMAPSPSLASSLLSSLSMDGLHGGTGSGSSSSPAASGML
6 AaTai  87.1%  17.5%   --KPNEPDFIMAIHTILNDNE-------------------VAMVESGLNNPSSSSSSLAIPSTSSGSGSSTARQMQQIT
7 MnTai  90.8%  19.5%   EGSQPEGSFIMATHTIIGPFQE----------GSGSSASDRLLPGSSSNSGSMLDETLGGRPGSTEGAGDDAG-------

            cov     pid   801       .         .         .         .         :         .         .         . 880
1 SgTai 100.0% 100.0%   -------------------------SVGGPLMSSAVNGQVSAVGSVGGRTNSSGFGSVTSDAN-----------SPAYHTY
2 LmTai  99.9%  87.1%   -------------------------SVGGPLMSSAVNGQVSAVGSVGGRTNSSGFGSVTSDAN-----------SPAYHTY
3 PkTai  84.4%  29.3%   -------------------------RLSAVTTPDNSNAMTLNNNNNNNSSSSNNNNNNNNNN------------SDYSAM
4 BgTai  90.8%  52.9%   -------------------------SLGGPLMSGPVNGQTAASSSAS-RVSSSGFGQLGSNSSSDTSNTITLNASSSYTTY
5 DmTai  92.7%  15.8%   PTHLLGGLVGGGQQGGGGNSTQTTSVGGPLMSSAIINGTGLQQQQQQQRSGASSSASSSAN---------------ALV
6 AaTai  87.1%  17.5%   --------------------QGVSNMGGPLMSSILNGSGNSGGPGGPSGLQGALGSVVSPRS---------------SNL
7 MnTai  90.8%  19.5%   --------------------VSSLSDNGRLRRLLSHSDSVGDLSEGPNKILKDLLNQKDEDD------------TQGGGV

            cov     pid   881       .        9         .         .         .         .         :         . 960
1 SgTai 100.0% 100.0%   ---STEIGLNDFD-FFPSSTWDIVGGGETSPDGSSVNAAQSGWDRRPDSRQSQSLTPVSTPNPRPPSGAGYSPSGGVCAS
2 LmTai  99.9%  87.1%   ---STEIGLNDFD-FFPSSTWDIVGAGETSPDGSSVNATQSGWERRPDSRQSQSLTPVSTPNPRPPSGAGYSPSGGVCAS
3 PkTai  84.4%  29.3%   S----DMSMHDFD-FFSRPDYDMEDR--------------TPWSDRPESRHS--LNSAPSPMFSHPSPAPNAPS------
4 BgTai  90.8%  52.9%   TLNSNELNLNEFD-LFPSSSTWDFGTSENSLDRAGAPQTPSAWGERPDSRQS--LTPVPTP--RPPSVPGYSPAGAMCPS
5 DmTai  92.7%  15.8%   NAFTASPAPAEHS-FYGSDTFEFDIAAHSSSFELDPSGGVGAWTDSRPNSRASVATPVSTP--RPPSGHGFSPAVCASPA
6 AaTai  87.1%  17.5%   HSSLLSQQSSDGS-SFFPSEFEFEFPHSTYDMES-----VGVNWDSRPDSRTSVT-PVSTP--RPPSVTAYSPAAASLCP
7 MnTai  90.8%  19.5%   GGGGGSGSLGRFIPHFSHLEKTPNASSSPSANSTHDNTMLKVLLNTDDDNENRLSSKDTDEIWNLLNTDENTSEFRQPQ

            cov     pid   961       .         .         .        0         .         .         .         . 1040
1 SgTai 100.0% 100.0%   PLNPYVHQTSP----AARVSTPAHPFANSFPFSPLQEQGPDGSFCSDGTEESKSGVQESAN-VNGSSDGGSAPGDSGRRL
2 LmTai  99.9%  87.1%   PLNPYVHQTSP----AARVSTPAHPFANSFPFSPLQEQGPDGSFCSDGTEESKSVVQESAN-VNGSSDSGSTPGDSGRRL
3 PkTai  84.4%  29.3%   -----------------------TPYGAPLPFSPLDN----------DMKDNKDMIMAEVNSINDSCES--------ARL
4 BgTai  90.8%  52.9%   PLNPYSAATQPSPAGTNRATTPANPFGNMLPFSPLQEQGPSAGFGLEETKDSKDGIVDMGNGVNVGEGSSNAPGELG-RL
5 DmTai  92.7%  15.8%   TPYQLSSHSAASL--PSPQSNASAGGGN------------YGGFNFHSFDSSDVKPEKDVQQQNQQQSNNNSSSSNPLL
6 AaTai  87.1%  17.5%   SPLTYQPSSAGGQ--PSPSNNQQVNNNNNNSSMTNNNAGLFGGFQCQFDDKD--KVQEQIQQQQQQLQQQQQQQQHQQQM
7 MnTai  90.8%  19.5%   GLGGGTQSTNS--------TSSLSSSHNTLSNNPQPTSNTVPPRPSSADLTSPNMLRGMKRPCDEAHDGNPSKQQQVGGF

            cov     pid  1041       :         .         .         .         .        1          .         . 1120
1 SgTai 100.0% 100.0%   RNLLLTKRQ---STSADEVSTGDAGGQVGDVDVRNRHRILKGLLNQDDD---EDARSDEVPAGAGAAGRGSVGGRLQADA
2 LmTai  99.9%  87.1%   RNLLLTKRQ---STSADEVSAADAGGQGGDVDVRNRHRILKGLLNQDDD---EESRGDEVPTGAGAAGRGSVGGRLQTDA
3 PkTai  84.4%  29.3%   RNLLTTKKS---NSHLDSEDQKNNRNKQILENLLNPP---EGVMDIDGA---DAG----PSPKQRRVTTPSSMNSNSDSS
4 BgTai  90.8%  52.9%   RNLLTNKRP---STGEEGNESIDHTGMGDVSDARNENRILKGLLNQDDK---DETRGDDSSSAMRSSPGSRHVGSRSTTS
5 DmTai  92.7%  15.8%   GGGLPNGVGGMLLSQQQQQQTPPQQQQQQQQESSERLRHLLTKSQSM---AGGLGGLGDDEKYFKPEGSEEEKHASGG
6 AaTai  87.1%  17.5%   ASHDSERLRNLLTTKRPHSNASSSSGMDMDHDHRNPNRILKGLLNSEED---KDSNGNKLSTTTLSQRLPQSVRAHQS--
7 MnTai  90.8%  19.5%   ESLLDPSPPASMSTIGQHGRPPTPHGGPLTPHGGPPTPHGRPGTPHGGPPTPHSGVGGPPTPIGNNSSSGMSHNGPGAPN

            cov     pid  1121       .         .         :         .         .         .         .        2 1200
1 SgTai 100.0% 100.0%   PRMSGHSSTGNNMLLKLLNEKSDDDDLEKRAGLKKQNELLQQLLKE--------QDEESKPQ-VGPDTG---QHRDDDPL
2 LmTai  99.9%  87.1%   PRMPGHSSTGNDMLLKLLNEKSDDDDVEKRAGLKKQNELLQQLLKE--------QDEESKPQ-VGPDTG---QHRDDDPL
3 PkTai  84.4%  29.3%   SSYQKSAGGSNTMLLTLLNKKPLPDSEDTD---TKTTEYLHQILKDGNGNQMMMEQELQHQQQVHQQLQSVHRIPNEDTI
4 BgTai  90.8%  52.9%   SEQPKVSSNNNNMLLKLLNEKSDDDDLEQ----QKQNELLQQLLLKK-------QDEERKPPPMSMDTGQQQQQQQEDPL
5 DmTai  92.7%  15.8%   FKMGGQPGGMGMFGPMGSMGRGVGNSSMLHKAGNSQNPMLLKLLNE--------KSEDDDGNGSGGGPGSMNNSRQSELM
6 AaTai  87.1%  17.5%   --QGGAVGGGGVGGGVVSAGLGGARPSTEHKSGS--NNMLLQLLND--------KSDDDDADAR---------NRPSELL
7 MnTai  90.8%  19.5%   NQGPSKLRERNRMLALLLAKESPINQSQNTPPTADPTGLPQEKLPKD-------LKEKMKVPGPWQGPGRHESQMLVELL

            cov     pid  1201       .         .         .         .         :         .         .         . 1280
1 SgTai 100.0% 100.0%   LESLGFRNPSPSPPSGAGVGVDVTGSLTVSSLIGGRKRPSDDGDESGVSSAKMRLG---GPSGAPPGVSRSGTAAGTSKL
2 LmTai  99.9%  87.1%   LESLGFRNPSPSPPSGAGVGVDVGGNLTVSSLIGGRKRASDEGDESGVSSAKIRLS---GPSGTPQGASRSGTAAGTSKL
3 PkTai  84.4%  29.3%   LEGIGFLSSPSSPPGG----------------SRLRKRPSEELDENGIPSKR--------NSKLDSIPPPPPVAIANNKL
4 BgTai  90.8%  52.9%   LKSLGFPNPTPSPPS----NTDPMTGIAASSLIGQRKRPSDDGDDGGPPSSKRLLDGMLNPTGSSVSSSGTTQPGGKSAL
5 DmTai  92.7%  15.8%   RQLKNPDGGSHGMHRN----------------SASGNMSTEDLKAMLKIQSDPSLNRKRSLNEPDDDPSAKRSEDKPSKL
6 AaTai  87.1%  17.5%   RQLQKVKDEPK---------------------PHQAPLKDEELLQMLQVQRN---DRKRSSTEPDEGAAAKRSDDKPSKL
7 MnTai  90.8%  19.5%   EKGHINNPPRPTLKAS----------------GPPLGMPVNPTVRTPGTPPIMVSPGGIGPPGLTQTSQPNMSTQGGMGM
```

```
              cov     pid 1201  .         .         .         .         :         .         .         . 1280
1 SgTai    100.0%  100.0%     LESLGFRNPSPSPPSGAGVGVDVTGSLTVSSLIGGRKRPSDDGDESGVSSAKMRLG---GPSGAPPGVSRSGTAAGTSKL
2 LmTai     99.9%   87.1%     LESLGFRNPSPSPPSGAGVGVDVGGNLTVSSLIGGRKRASDEGDESGVSSAKIRLS---GPSGTPQGASRSGTAAGTSKL
3 PkTai     84.4%   29.3%     LEGIGFLSSPSSPPGG----------------SRLRKRPSEELDENGIPSKR--------NSKLDSIPPPPPVAIANNKL
4 BgTai     90.8%   52.9%     LKSLGFPNPTPSPPS----NTDPMTGIAASSLIGQRKRPSDDGDDGGPPSSKRLLDGMLNPTGSSVSSSGTTQPGGKSAL
5 DmTai     92.7%   15.8%     RQLKNPDGGSHGMHRN----------------SASGNMSTEDLKAMLKIQSDPSLNRKRSLNEPDDDPSAKRSEDKPSKL
6 AaTai     87.1%   17.5%     RQLQKVKDEPK---------------------PHQAPLKDEELLQMLQVQRN---DRKRSSTEPDEGAAAKRSDDKPSKL
7 MnTai     90.8%   19.5%     EKGHINNPPRPTLKAS----------------GPPLGMPVNPTVRTPGTPPIMVSPGGIGPPGLTQTSQPNMSTQGGMGM

              cov     pid 1281  .         3         .         .         .         .         :         . 1360
1 SgTai    100.0%  100.0%     WERNKMLASLLAKEPSQ-PATIPPIPASVISATPQEILPRVSKPQTP---SWPSGNVQPPSSTAATARPTQQVTDVSRQQ
2 LmTai     99.9%   87.1%     WERNKMLASLLAKEPSQ-PATIPPIPASVISATPQEILPRVSKPQTP---SWPSGNAQPPSSTAAPSRPTQQVTDVSRQQ
3 PkTai     84.4%   29.3%     REKNKTLLQLLAKDPPKKPEPIPHLPPSVISAKPQDRLPKVVITTAS-------SASRPNTSVWATSNS-----------
4 BgTai     90.8%   52.9%     WEKNKMLASLLAKQPSK-TATIPPIPASIITAMPQEKLPRVVDPSKSRPGGWSGGGLQPPSTTQSATRP-----------
5 DmTai     92.7%   15.8%     CTQNKMLAKLLQ-NPPKIPKAPNPEQPLQVKTLPDITSSTVSSTLAAPGNLISAGSTG--PKAAANRNR-----------
6 AaTai     87.1%   17.5%     REKNKMLASLLA-NPAKAPTQMLAG-HSSLRIIPDIPTSNISRQMGSVTSSTAPNQTLNNNNLTTSNNN-----------
7 MnTai     90.8%   19.5%     NNNNNMCPGVSNRVSMSGSFVSTTSTNSLPSITDSFNISNQVSSCYPEGSLPPMPIITSSAASFGSQND-----------

              cov     pid 1361  .         .         .         4         .         .         .         . 1440
1 SgTai    100.0%  100.0%     VRPQPNRPSATPFLNQMLTQQDHQQSNMLLQQSIQQQKVQQQPSQQQQQQQQAQQQQVQSSQQQAQQQVQQQQQQQQQQQ
2 LmTai     99.9%   87.1%     VRPQSNRQSATPFLNQMLTQQDHQQSNLLLQQSIQQQKVQQQQQQQQPSPQQSQQQQVQSSPQQAQHQVQQQQQQQQQQQ
3 PkTai     84.4%   29.3%     ----------------------------------QQQQQQLPGQIRMTPQQQRAPTSNPPTRGGFLNELLQSSQQQQQMQ
4 BgTai     90.8%   52.9%     -------------------------------------NLQQQAQVRRPAPNFPVN-------HPDQHQVLLQHQKQQQQQ
5 DmTai     92.7%   15.8%     ------------------------------------KQQQQQQQQQQQQQQQQVAGIPSQQQQQQPNDVYLSQQQQQQLQ
6 AaTai     87.1%   17.5%     ------------------------------------LKHVQHLRMQQQQQMRKSAMPSPSQQPPTSSDIYLSHQQQQQAL
7 MnTai     90.8%   19.5%     ------------------------------------SESQLRESVINDILEMDNDEVRINSSPLNMDDLHQIPNSSADAE

              cov     pid 1441  :         .         .         .         .         5         .         . 1520
1 SgTai    100.0%  100.0%     QQQ---VQQQRGQLNQLESSFNIPVSGATTVDFRSVSGSGVSSPSTWDNQSSDPVLSDLLD-----QVIDIVPD----DS
2 LmTai     99.9%   87.1%     QQQQQQVQQQRAQLNQLDSSFNLPVSGTTTVDFRTAPGSGVSSPSTWDNQSSDPVLSDLLD-----QVIDIVPD----DS
3 PkTai     84.4%   29.3%     QQQLQLPHQLRDQMRISNNNNILPWDSHNSDDHDLNKLLDQLFENAPDAISEDPNLLGLIQSVAQGDVMNSTDNSMGVNN
4 BgTai     90.8%   52.9%     LQQ-----QQQAATAAYITGWNL----CYTSEFRAGLVGASDSPSMWDGQSSDPVLSELLD-----QVIDIVPEAIISDN
5 DmTai     92.7%   15.8%     SPQLAFQHQQLATTATTSITTAAS------TSAAAAAAAAILGEGDSELSKLLDSVMEYYP-----DDTPIVTNAPSEAS
6 AaTai     87.1%   17.5%     LQQQLIAAQQRQQNYAAAIQDSGIGSVGSGPFATPSTATSTTSHATPEWDPELNEILNHVI-----DIAPDVSFADSELT
7 MnTai     90.8%   19.5%     IQKIEKSLMYAAETQSSLVISEAGSLPPYTAVVSASSMSNYPPPYNHKRIPNAGPPGGPGG------GTGMVGSHMNAGG

              cov     pid 1521  .         .         :         .         .         .         .       6 1600
1 SgTai    100.0%  100.0%     SAIMNMLD------AMES-SPNNSGYQQGLNEKMAIHAIQKSLMLCESAVKSPSSPTVSLPGTPPTYAASTMSNQTPQQG
2 LmTai     99.9%   87.1%     SAIMNMLD------AMES-SPNNSGFQQGLNEKMAIHAIQKSLMLCESAVKSPTSPTVSLPGTPPTYAASTMSNQTPQQG
3 PkTai     84.4%   29.3%     VTLNNVIDSSNEKMAIHAIKNSFMSYEQQATISNRMQQQINQSVLLQHQQQQQQQLQQQQQNQQAAQQQKQAHLTMNQSG
4 BgTai     90.8%   52.9%     ATLMNILD------AIEPPTSSTSSFQQDISEKMAISAIQKSLMQCETVMKSPSSPTISLPGTPPAYTSTAVS----QQG
5 DmTai     92.7%   15.8%     AINDIQKSLMLDVESAAFGNDLNQQLMMTQQQQHQQQQQQQQLLALQLAQQQQQQRQQHLQQPPAYPGMLNMQQQQHQQQ
6 AaTai     87.1%   17.5%     SFLGSMDPSTSTAPSQASQQDIQEKLAINAIQKSLMREIENVTSPMQYSGS-----------PPAYP-MHGMGSVGSPAG
7 MnTai     90.8%   19.5%     MMRPTIQFSPNPGSPTIPSRPSLPPQRMNANLTQRSQEYLRQRLLQQQQQQVIVPSNTSDVTQPPASSYQAMNMDDLLNN

              cov     pid 1601  .         .         .         .         :         .         .         . 1680
1 SgTai    100.0%  100.0%     S-FPPPPVYQRP--RFGLPAGTTPVPGRP-GAPQYGIGVLN----------QQALIQQRKILQQQQQ--KQRLLQQQQQQ
2 LmTai     99.9%   87.1%     N-FPPPPVYQRPT-RFGLPAGTTPVPGRP-GAPQYGIGVLNQQALGSGVLIQQALIQQRKILQQQQQ--KQRLLQQQQQQ
3 PkTai     84.4%   29.3%     QPFPPPPMYQQRPNRLQTHQNSVPQLGNRPMTQQQQFAVATSTG-----QFSPAQVSQAQNHARAMHMQQKRLLMLQQHQ
4 BgTai     90.8%   52.9%     G-FPPPPLYQQPRTRLNIPQNAGQLGVRPAGAIPYTNSQLTSQ--------QQMLMHQKRILQQQQQQQKQRLLQQQQQQ
5 DmTai     92.7%   15.8%     QQQNQQHIMQRLEAMRNQGNQGFQRPPPMYPARGRGPMNAVATPGGVVLPAQQQLRNIRQQQQLAAAQQKERLLQQQQKQ
6 AaTai     87.1%   17.5%     QMSQQTPGTPTTPNPNFTPPPVYQPRLRMSGPGGPGVGQLGNGAPSPSAIAMQKLSHQQQQRERMQEQQRQRLLQQQKQQ
7 MnTai     90.8%   19.5%     SIAPNVNVTLQRNVADSQTSPSYNLLNSPLSGGQISPGQRIPQP-----PYSPHGQTTSPIPNQQYTNSGQMSGYQTPQG

              cov     pid 1681  .         7         .         .         .         .         :         . 1760
1 SgTai    100.0%  100.0%     QLLIPSNATADQ-----LPTGLQNIDSLLNNTVAPNVSLQRSASVP-----DSQLSPNFGGQVLNS----AASQISPGQR
2 LmTai     99.9%   87.1%     QLLIPSNATADQ-----LPTGLQNIDSLLNNTVAPNVSLQRSASVPA----DPQLSPNYGGQILNS----AASQISPG-R
3 PkTai     84.4%   29.3%     MVIPSNAATNSQ-----GTTSLQNIDSLLNNAAPPNVSLQRSTSLTSEGSNAQQLSPGGSNNPLYNNTMLTSSQISPP--
4 BgTai     90.8%   52.9%     QLLIPNNATAAD-----LPSNLQNIDSLLNNSVAPNVALQRSSSVP-----DXQLSPNYGGQMLNS----ASSQISPG--
5 DmTai     92.7%   15.8%     QLLVPENATG-------MNAGLNNIGSLLNTTGAPNVSLSRTN-LPSD----AQLSPNFAQT-LMQQQLSPGRSAPYSPQ
6 AaTai     87.1%   17.5%     QMVVPVNATANADLNLGLTPGMQNIESLLNNTVAPNVSLTRANSVVPD----SQLSPGFSPSQLMQQQLSPNQRTQLSPQ
7 MnTai     90.8%   19.5%     SQLSPRLSQGSP-----VPSSYQPGGQPLPQMSPTGAPGTPVVQVPSG-----QQSPAWSAHSPQPR---PSLMIQNPIL

              cov     pid 1761  .         .         .         8         .         .         .         . 1840
1 SgTai    100.0%  100.0%     LNQQPYSPHSQLASPLG--------------------QQQVFSPVSTGMNSFSSGQQAAQSRLSPHPPTGLPSFQQSQLS
2 LmTai     99.9%   87.1%     LNQQPYSPHSQLASPLG--------------------QQQVFSTVSTGINSFNSGQQAAQSRLSPHPPTGLPSFQQSQLS
3 PkTai     84.4%   29.3%     -QRQPPSSLASPYSPLSTN-----------------HQQQSFSNLN--FTGASNQQAVAQSQGARLSPSGLSPFQ-AQLS
4 BgTai     90.8%   52.9%     -QRQPYSPHSQLTSPLGQAQSQTQQQQQQQQQQQQQQQQQGFSPVGSGLNNFTNNQQGAQARLSPHPPGGLPPFQQSQLS
5 DmTai     92.7%   15.8%     PNQGYAPQFPQ-PGQRLSP------------------QQQQQLSQQQQNNVQQQQLAYQQQQVGDGGRSNTPFGSNSGMQ
6 AaTai     87.1%   17.5%     QTGFQGNPFNNNPGHRMSP------------------QQQQQLVAAAG---FQQGNPGNQQLSPRQPPFNTSQQLPQANA
7 MnTai     90.8%   19.5%     NAQLGGSYMRPDNRPYPSG-------------------VRGGQLPSMRSMPSPGTRHSPYPSMGPPTPGSTAQGDAGPYP
```

```
                 cov    pid 1841          :         .         .         .         .         9         .         . 1920
 1 SgTai 100.0% 100.0%     PRISQGQPGYPVLTQ-----------------------------------------------------------------
 2 LmTai  99.9%  87.1%     PRISQGQPGYPVLTQGTLAQSPQGQQTVSSWSQAASRLSLQQQQQQQQQQQQQQQQQQQQQQQQQQQQQQQQQQQQNPVL
 3 PkTai  84.4%  29.3%     PRVSQPQTTFTGTSVMASG-------------------------AQQSQMQQQSNWASQQPQQSMVNNRMSLQQQQNPML
 4 BgTai  90.8%  52.9%     PRISQGQQAYPALSN--------------------------------------SLTQSSQAASNWTQSRHLQQQQQNPLL
 5 DmTai  92.7%  15.8%     SPGMQNSPQQWGSGGGGGG---------------------------------------GPGGPLPSGNAGRTLQQHNPML
 6 AaTai  87.1%  17.5%     AALQQHQQQQWQQN------------------------------------------------------ARLSIQQQNPML
 7 MnTai  90.8%  19.5%     PSSPQGQPSSMYQQQ------------------------------------------------QPQQPTQFRLQRAGPMA

                 cov    pid 1921          .         .         :         .         .         .         .         0 2000
 1 SgTai 100.0% 100.0%     -----------GRPFGPQRQQQQQQPSQQQQPQQLPPVRSLASPGGAVVGPRQSPYQAEQFPPPSSPGTTTGFQAQGQFP
 2 LmTai  99.9%  87.1%     NAQLSGNYGTAGRPFAPQRPQQQQPSQQQQQPQQLPPVRSLASPGGAVVGPRQSPYQAEQFPPPSSPGTTTGFQAQGQFP
 3 PkTai  84.4%  29.3%     DARLSG-----GVYTGNRAALFAQQSQKSQQAQ--SPQLSQVRGGLTSPGPRQSPFPADISPTTATSYAAASHG------
 4 BgTai  90.8%  52.9%     NAQLT------GSYGGGGGRQFTQRGQQATTQQQLSSVRSLPSPG-TPGGPRQSPYPPESFPPPASP-TAGNYQTGGQFP
 5 DmTai  92.7%  15.8%     IAQLQGVSP-YNARQYQQNQRRGLNSPGAVGPGGNPAAQAALQRQNSFQGQGGGGATTPDGSGVGFGGPQSPYGTNVNVF
 6 AaTai  87.1%  17.5%     NAQLSTVPG-FNPAAAANRQFVAPQRQRSLNSPGTPRQGSFG---STVDGSGFPGPPSPSQPNAGAGPNFG------NPV
 7 MnTai  90.8%  19.5%     GVHSPRSAHHFPPAPGQHHDPSAPPPHLSPAAGGHLPSPHSVPPSALAHHPHMGHHHPGSMPPSLMASVGSS--------

                 cov    pid 2001          .         .         .         .         :         .         .         . 2080
 1 SgTai 100.0% 100.0%     QQLRLQRTISAPS---ATTQLPGGVNHSRLYGTKQELP-HP-ALSPSEPVSPHPFHPHGPPLPDPHHHHPHPLSHPHPHH
 2 LmTai  99.9%  87.1%     QQLRLQRTISAPS---ATTQLPGGVNHSRHYGTKQELP-HP-ALSPSEPVSPHPFHPHGPPLPDPHHHHPHPLSHPHPHH
 3 PkTai  84.4%  29.3%     -QYRLQRTMSVPNSNMAASQVPPGNNCIRAYSSNTGAS------LNFKDTSQTPPHPPPLYSPVMYQHHG----------
 4 BgTai  90.8%  52.9%     QQLRLQRTISAPS---ATTQLPGGVNSPRPYGLKQDPHSHPHLLSPSHPMSPHPFHPPDPHSLQQHHHHP----------
 5 DmTai  92.7%  15.8%     QQQQLQRLQRQGSVP-QATQHLPGSPRFGSSPGSSNNTDSSAAAGNQQQQQQQQQQQQQQHQQQQHQQLMNGLSMSPHPH
 6 AaTai  87.1%  17.5%     FANQQMRLQRQGSVPPQATQHLPGSPRP-SYGGHGPGPDTSGYGMMFNNATMQQQHS-----------------------
 7 MnTai  90.8%  19.5%     -PMGCPSVVSSAGGPGDVGPHMVGGAAMGGGGGAGGSGMPGMVGGVMSQSYGGEHAPPHPHSYEAAAQYYG---------

                 cov    pid 2081          .         1         .         .         .         .         :         . 2160
 1 SgTai 100.0% 100.0%     HHHHIPHHQQQPQPQHHNPHQPSQHHPVMYQAQPRPPPDSQFCYDQPNFQLYGASERGRPQAHTPTNHQPGGGGGGGG--
 2 LmTai  99.9%  87.1%     HHHHIPHHQQQPQPQHHNPHQPSQHHPVMYQAQPRPPPDSQFCYDQPNFQLYGASERGRPQAHTPTNHQPGGGAGGGG--
 3 PkTai  84.4%  29.3%     -------------------------------------ESSPYCYPDQSLPMYGDRSRVTHPSGGNNNVNNNNN-------
 4 BgTai  90.8%  52.9%     -----------------------QHHPMLYHP----PPDSQFCFTQGELPLYGANDRGRPQAHTPTNHQPGGG-------
 5 DmTai  92.7%  15.8%     PH-------------------PAMMGVGGMGSGGGNGIIGLGGGGGNYGATLGGYGQSQQANDFYGRAQTAGGGGSVA--
 6 AaTai  87.1%  17.5%     -----------------------------AGSPGDYFNRSQAGGNNGGGLGTSEHVRQELRAVVSGRAQNAASGGVGG--
 7 MnTai  90.8%  19.5%     -------------------------------------PPDPANRNSTGGNGMLSEYTRNELRAHVGARSTGGGGGGPASP

                 cov    pid 2161          .         .         .         2         .         .         .         . 2240
 1 SgTai 100.0% 100.0%     NGGMTSEYVRQELRAIVGARQSNGGSSGGVGMTVSGQQRQLQLGPGQSVSAADLEAFG----FVCEPPSTGTSDSPKLWG
 2 LmTai  99.9%  87.1%     NGGMTSEYVRQELRAIVGARQSNGGSSGSVGMAVSGQQRQLQLGPGQSVSAADLEALG----FVCEPPSTGTSDSPKLWG
 3 PkTai  84.4%  29.3%     NNNGVNEFVRRELRTVVTARQQQGDMR----------LPPLQVGPGQQTIATGLEHLG----LSLEMP----NDDDNKWG
 4 BgTai  90.8%  52.9%     NNGISSEYVRQELRAVVGARTQQQSGAGGQNQGTR-QQQQAQLQLGQQVSPTDLDPLGT---LSFEIPTSGASDSKQLWG
 5 DmTai  92.7%  15.8%     GGNANSEFVKQELRAVVSVRAQQAAAAATGGAGVGGGVAQRGQTPQSPLQQGSVIGGGGGIVGGVNSTNTMGNVTPTGSG
 6 AaTai  87.1%  17.5%     AG-------------GLRTPQSPIGMSPMGGVGVGGSGAGAGTGPGG---GGSGAGSGGGVMTGMGGANNVGSTNSAGMG
 7 MnTai  90.8%  19.5%     SPSQQQQPPQQQQQPQPQPQQQQQQQQQQQQQQQSQQQQQHHHHPQQQPQQQQQQHQQQQQHQTSPPPHLAFQQPPDHHH

                 cov    pid 2241          :         .         .         .         .         3         .         . 2320
 1 SgTai 100.0% 100.0%     SIGSELSSGSPQAGFSSSRNPTEE-----------------SPRPSDHKSSLLQKLLSES--------------------
 2 LmTai  99.9%  87.1%     GIGSDLSSGSPQAGFSSSRNPTEE-----------------SPRPGDHKSSLLQKLLSEVTLSSAAGGIGSNLLSGLHHG
 3 PkTai  84.4%  29.3%     GISNDMTEGSPSAGFSVRTSLDDVNLMRSGVGNNANAMNQMGNVGGGEKASLLQKLLSE---------------------
 4 BgTai  90.8%  52.9%     NIG----SGSPQPGLSSSRNTMEE-----------------PPRPGDQKSSLLQKLLSE---------------------
 5 DmTai  92.7%  15.8%     NVSNSMLNTPPDPTLSFSFETPDFFTSSAT----------RMSIAAAENAGLSPSDLPDHWASGTSNPSTSTSSSSSSSI
 6 AaTai  87.1%  17.5%     PGSNGSVLSPGMGGITGNLVGSGGGVGGSG----------TGSVGGGGGGGVNSSGNGDGMQT-SQLPGTSSLLNTPGDI
 7 MnTai  90.8%  19.5%     HHHQHHLEQPLLHSYSPSDNNNWAG----------------ESVNTTQAGALSGSPRMDDNRGNTGSEKGKTESMLKHLL

                 cov    pid 2321          .         .         :         .        ] 2369
 1 SgTai 100.0% 100.0%     -------------------------------------------------
 2 LmTai  99.9%  87.1%     LAPGTHPLQRATAASGGGAGAGGVAGASTAAALTLQQHQHQQQQQQQQQ
 3 PkTai  84.4%  29.3%     -------------------------------------------------
 4 BgTai  90.8%  52.9%     -------------------------------------------------
 5 DmTai  92.7%  15.8%     SASGSNNNKYNSGNISVCNLPRSSPAAATAA------------------
 6 AaTai  87.1%  17.5%     DPSMLFNFHLAPKEFFGGNTSRAP-------------------------
 7 MnTai  90.8%  19.5%     SKMNIP-------------------------------------------
```
