# Supplementary material for: Crucial Role of Juvenile Hormone Receptor Components Methoprene-Tolerant and Taiman in Sexual Maturation of Adult Male Desert Locusts
Source: Biomolecules. 2021 Feb 9;11(2):244. doi: 10.3390/biom11020244 (PMC7915749; doi:10.3390/biom11020244)
Supplement: Supplementary file 1 [file biomolecules-11-00244-s001.zip › SupplementaryFiles/SuppFig9.pdf]

>MK855050.1 Schistocerca gregaria methoprene-tolerant protein (Met) mRNA, partial cds

GGCCGGCCAGGGCGCCGAGCAGACGACGTACGAGCTGGTTCACTTCCTCGGCTACCTGCAGGTCCCGCGG
TCACACGAGCCGCCGCCCGCCACGCACCGCCCGCGCCGACACCGCGACACAGTTCCAAGTAGCAACAGTG
ATAATGTCCTGGTTGCTGTCGTCCAACTGTTTCGGGAGAAAAAAGTCATGGAGTTATCTCTCCTGGAGGC
AACACTGGATGAGTACCAAACGAGGCATCTAAAAGATGGCAGTATTGTGTATTCAGATCACAGAATATCT
GTTGTGGCCGGATATCTGGCAGATGAGGTTCACGGTCATAATGCATTCACGTTTATGCATCAGGATGACA
TGGCATACGCCTTAATAGCCCTTCAGCAGATGTTTGACAAGAATGAGCCGTTTGGCAGTTCCACTTACAG
GCTCGTTACAAAAAATGGGCAATACATTTATATGAGAACAAGAGGCTATTTAGAGTTTGCACATGGATCA
AAAGATGTGGAGACATTTTTGTGCATTAACACACTTCTGTCGAAAGAAGAAGGTGAAGATGGGCTGCGGG
AGATGAAGCAGCGCTTCTCTGCTTATGTCTCTGCACTGAGAGCTGCTGGAGCTGACTTGTCCTTGGCTGG
CATTGCCGCTGCTATGAGTGCTGCACCGCCCCCACCCCGGATGGTGAAAGGTTCTGAGCTTCCCAAACTG
CCACCCCCACCCCCGCCCCCACCACCATCTTTGCAGCCATCATCGCCATCACCACTGCCACCACCACGGC
CACCACCACCACCACCACCACAACCACCTGCAGCAATCACGTCATTGTCATCGCCTGAGATTATAACTAA
TTCAACTCTCTGTGAAGTGTCTCCGCCCTTCACAGCAATGGTGACAGAGAGTGCAGCTAGCTCAGAGCAG
AGTGCAGATGTCTTTGGCAGTGTTTCACGCTCAGAAGCAGCAGCAAGCAAATACAGAAACCAAGCACCAA
CAGCAGCTTCCAGATCACCTACAGTGGATCCATATCTTCCAGCAGCAAGACCTCGTGCAGTTTCTGAAGG
AGATGACACTACTGGATTCAATTTACAGAATTTTGTAAGTCTCTTCAAGGTGCCTGAAGAGGAAGAAAAT
AGTGTTGAAGTTCAGCTGTCACCTCTTGCCAATAGTACTGGCATTACCACAGACTTAGAAGAAAAAGAAC
AGCTCAGAAATCCACTTTCTCCTTCATCACCTCCATCGAGGCACCCATACACCGCTGCCCAGAGGATTAG
GGCAGCATCAGAAAGTGAACTACAGACTGCCAATAGTGGATCGACTTTTATTCAGTCAGATCCAGCGAGC
AGAGGGGGCAACTGGCAGTTTCCGAGGCCCGGAGACATGCCTCATTCGGGAACTACGGAGAGAAGGCCCC
CGGAAAGACCTACGGTTATTACCGGACGGTGCTGTGCTGGATCTGAGAGGATGAAGTGCAGTAGATTGTC
AGTTATGTCAGTCACAGGAGATGAGAAGAGGATTACCAGTGTGCGTGGTGAAGTTACTGTGTTAAAAAGG
ACTATGAGTGCAGATGTAGAAAATCCGAGCACTACAAAGAAACTGAATGTTGGACCCCGGTACTGTAGGC
AGCGGCACAGAGTTGAGCATCCTCCTACGGAAGCTCTGAGTGTGGAAGTAAATACACTTCCGCCCGATGA
ACTGCAGCATATAATATTGTCGAGGGACAGTGGCATGTACAATTCGCAGCCAACAGTTCTCGTATCACAG
CACAGCCTGTTTCGGACATCACAGCCAGCACTTCAGACTGTGACACAAAATGTGCCTGCATCTTCTTTGA
GCCCCCCTCAATTAGCCGAAATGCAGAGCACGTCTGTTACACCACCATCGTTTATTATGGAAGAACCTAG
TGGCCTGCCTCTTGTGAACTTAAGTGATGCCAACACCTTATCAGTTGGTTGCCAGGATCCTCTTATCAGC
CCTGATCATGGAGACAGTGACTCATTTTCATCTCTAGGATTACCTGATCCAGAGAATAATCCAGAGGTCT
TAGACCTGTTAGAAGCATCTGAACTGAAGCTAAATTTTGACACATTGCAAGAGGTGCGCAGTAGCAACGA
ACCAGAAGCACCTTCCGGACCGGAGACTGCAGTCACTCTGGAGCGCTTTCTGGCACGCAAACACCTACAA
CTCGAGTCGTGCATGCAGCGACAGGAAACACAGATGCACTGCATCGAGCACGACCTGACTCAGCTCCCAT
TGGCAGACCATGTGTTCCGCTCAAACCTCACACAACTGCAGGCAAGTGGCCCGATACATGCCAGGAAGGA
AGAGAGACAAGCTTCAAAATTACACATTCCTGTCCTCACACCTATATTTATGAGGAACAACATAAGAAGC
ACCAGATACTGCTGA
