# Supplementary figures and images for: Crucial Role of Juvenile Hormone Receptor Components Methoprene-Tolerant and Taiman in Sexual Maturation of Adult Male Desert Locusts
Source: Biomolecules. 2021 Feb 9;11(2):244. doi: 10.3390/biom11020244 (PMC7915749; doi:10.3390/biom11020244)

Tree scale: 1

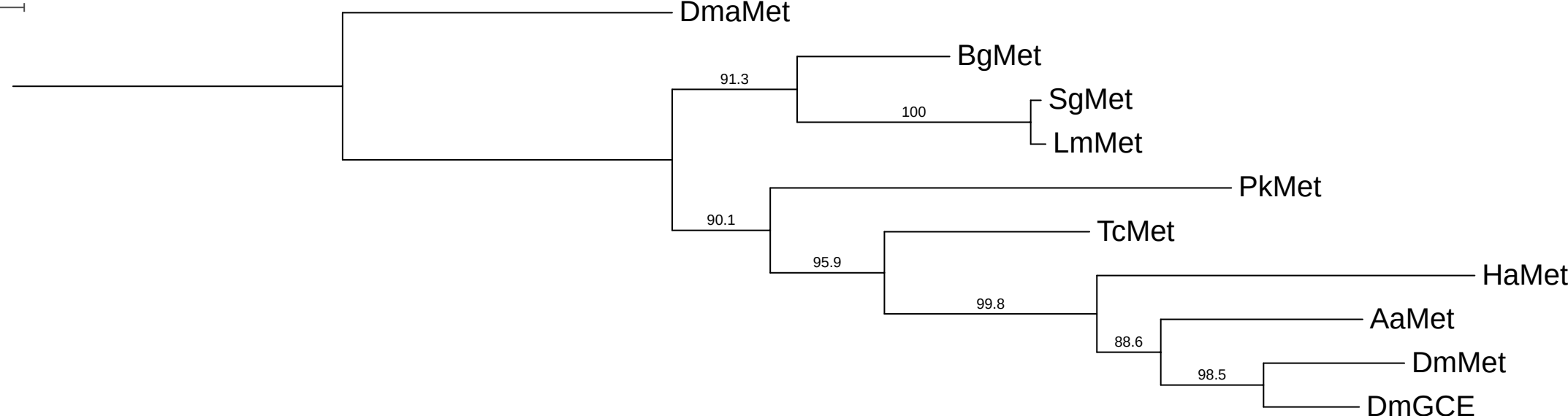

Supplement: Supplementary file 1 [file biomolecules-11-00244-s001.zip › SupplementaryFiles/SuppFig3.pdf]

Tree scale: 1

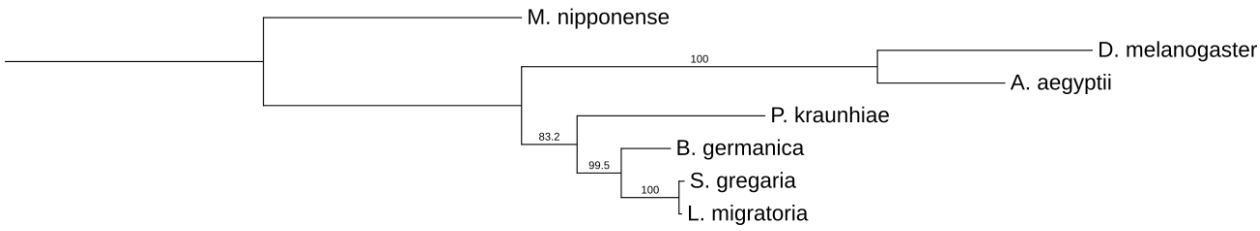

Supplement: Supplementary file 1 [file biomolecules-11-00244-s001.zip › SupplementaryFiles/SuppFig6.pdf]

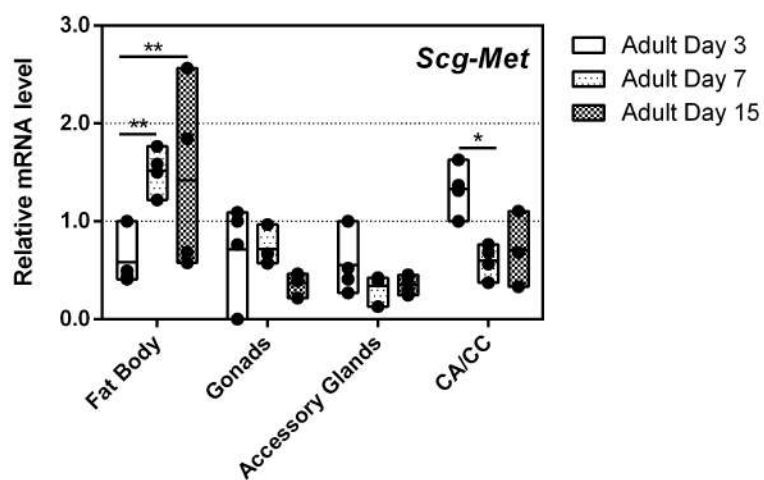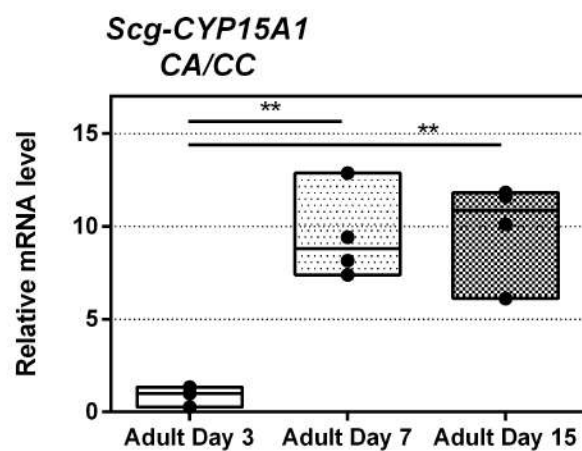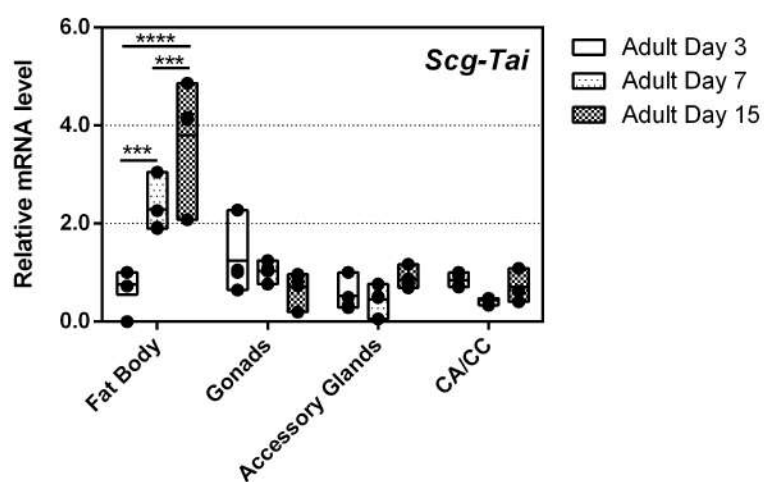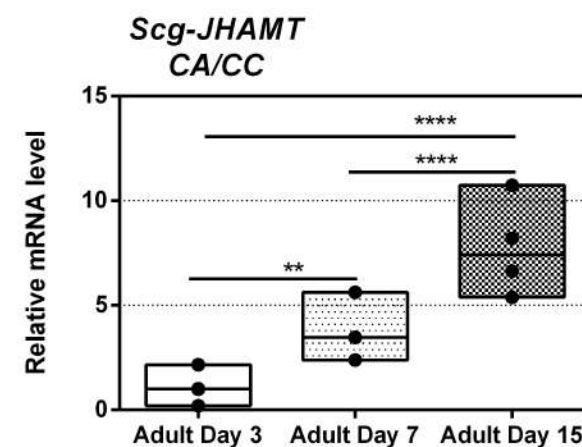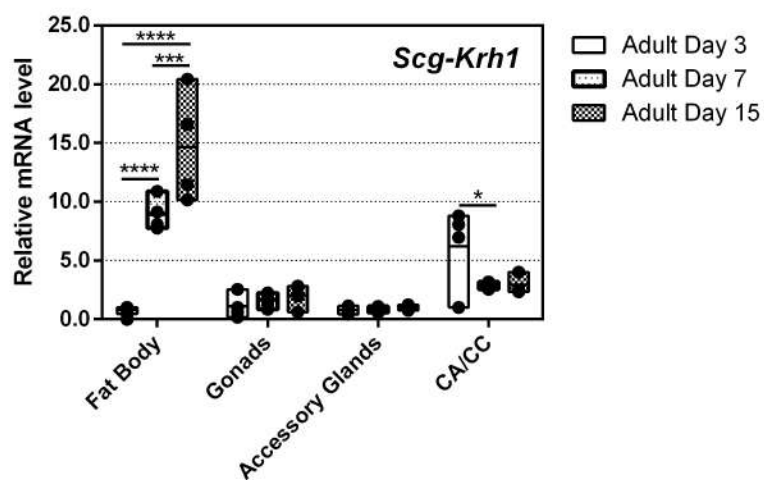

Supplement: Supplementary file 1 [file biomolecules-11-00244-s001.zip › SupplementaryFiles/SuppFig7.pdf]

Control

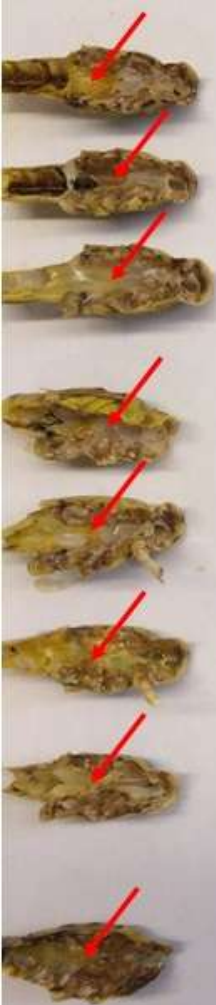

ds Mek

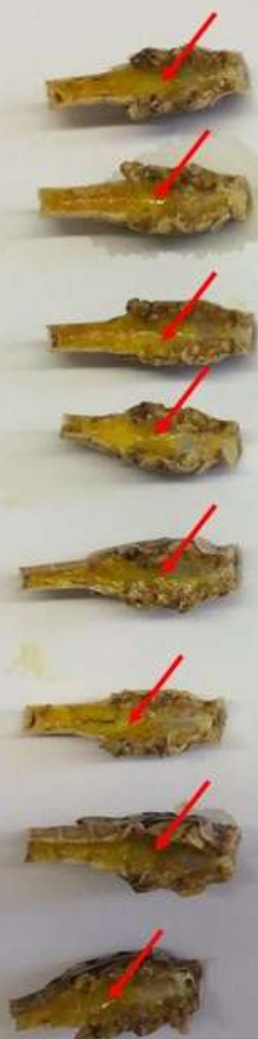

ds Tai

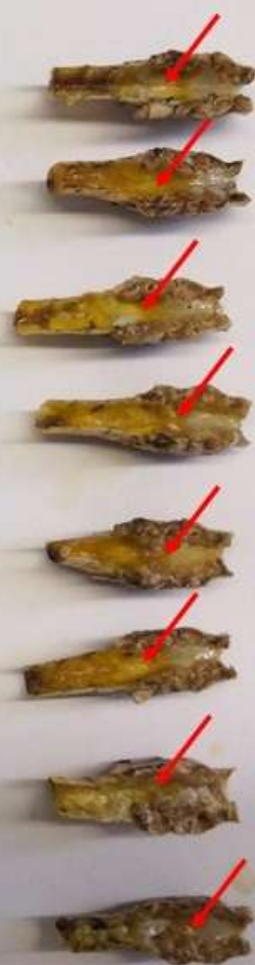

Supplement: Supplementary file 1 [file biomolecules-11-00244-s001.zip › SupplementaryFiles/SuppFig8.pdf]
